# TMEM135 deficiency prevents intestinal lipid accumulation by coordinating lipid uptake and oxidation through ELOVL6-mediated PPARα activation

**DOI:** 10.1038/s12276-026-01795-z

**Published:** 2026-08-05

**Authors:** Hyunsoo Kim, Channy Park, Xiaofan Wei, Arun Chhetri, Laxman Manandhar, Jiwon Seo, Yoonkyung Park, Sang-Wook Lee, Raekil Park

**Affiliations:** 1 Department of Biomedical Science and Engineering, Gwangju Institute of Science and Technology, Gwangju, Republic of Korea; 2 Department of Chemistry, Gwangju Institute of Science and Technology, Gwangju, Republic of Korea; 3 Department of Biomedical Science, Chosun University, Gwangju, Republic of Korea; 4 Department of Radiation Oncology, Asan Medical Center, Seoul, Republic of Korea

**Keywords:** Fat metabolism, Homeostasis

## Abstract

Excessive lipid accumulation impairs digestion and gastrointestinal function. Transmembrane protein 135 (TMEM135) is involved in lipid metabolism of various tissues. Here, TMEM135 deficiency increased intestinal lipid absorption through ELOVL6-dependent oleoylethanolamide production and subsequent activation of peroxisome proliferator-activated receptor alpha (PPARα), which upregulated CD36 at the plasma membrane in the intestine of mice fed a high-fat diet or lard oil. Despite this enhanced lipid uptake, long-term lipid accumulation was suppressed by elevated peroxisomal and mitochondrial β-oxidation through increased fatty acid oxidation and oxidative phosphorylation activities without chylomicron secretion in TMEM135-depleted intestine. Inhibition of PPARα or ELOVL6 attenuated these effects. Furthermore, consistent results were observed in both TMEM135 KO and intestine-specific TMEM135^iKO^ mice, confirming tissue-specific rather than systemic effects. Overall, these results provide insight into the role of TMEM135 as a regulatory node connecting intestinal lipid absorption and systemic energy expenditure, contributing to the coordination between lipid storage and oxidation during metabolic adaptation.

**Figure:**
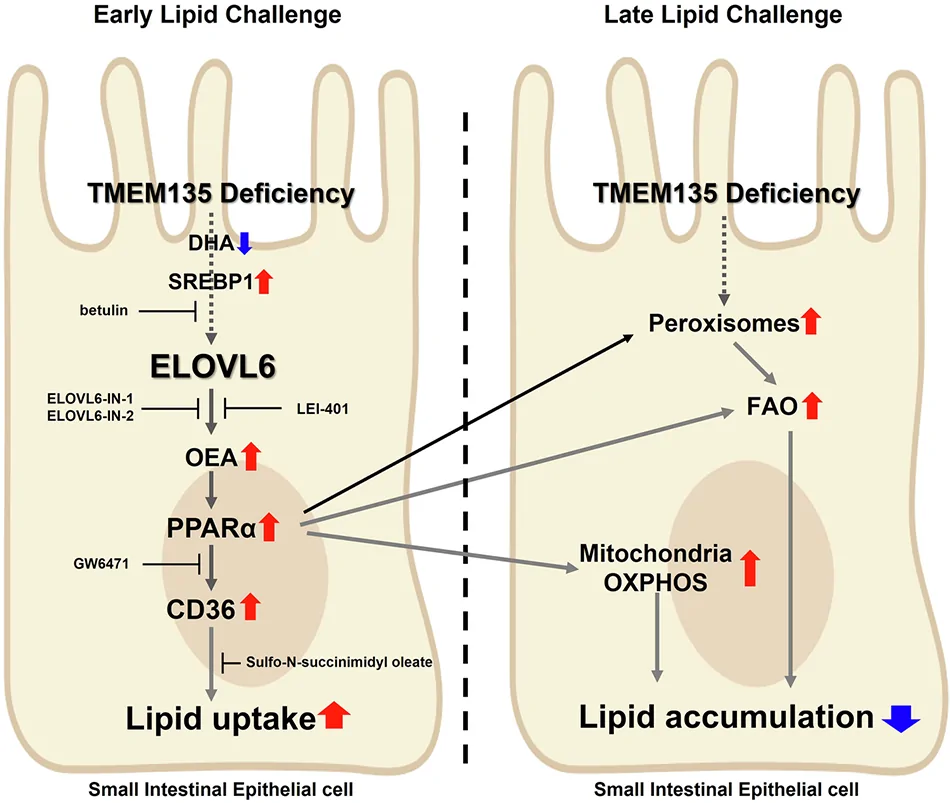

## TMEM135 links intestinal lipid absorption and energy expenditure

Intestinal lipid metabolism has a crucial role in metabolic diseases, with transmembrane protein 135 (TMEM135) emerging as a key player in this process. This study reveals that TMEM135 deficiency enhances lipid absorption and utilization in the intestine, addressing the previously unexplored role of TMEM135 in intestinal lipid metabolism. Using CRISPR–Cas9 technology, researchers created TMEM135 KO mice to study lipid uptake and oxidation. They found that TMEM135 deficiency increases long-chain fatty acid uptake without causing lipid accumulation, owing to enhanced lipid oxidation. This process is mediated by CD36, a lipid transporter, and peroxisome proliferator-activated receptor alpha, a transcription factor activated by ELOVL6 and oleoylethanolamide. The study highlights the importance of TMEM135 in maintaining lipid homeostasis and suggests potential therapeutic targets for metabolic diseases. Future research could explore the role of TMEM135 in other tissues and its potential as a therapeutic target. This summary was initially drafted using artificial intelligence, then revised and fact-checked by the author.

## Introduction

Intestinal lipid metabolism is critical in metabolic diseases^1^. The decline in lipid utilization processes, including fatty acid oxidation (FAO), lipolysis, lipophagy, or chylomicron secretion, leads to the accumulation of cytoplasmic lipid droplets (LDs)^2^. The abundance of cytoplasmic LDs is increased in intestinal epithelial cells of diet-induced obesity and *ob/ob* mouse models^2^. Impaired chylomicron secretion by GRASP55 deficiency promotes intestinal lipid accumulation in lipid challenge^3^. Intestinal lipid accumulation of patients who are obese impairs digestive function and worsens gastrointestinal symptoms such as diarrhea, abdominal pain, and incomplete evacuation^1^. However, intestinal lipids utilization and accumulation have not been sufficiently investigated in this context.

Transmembrane protein 135 (TMEM135) is located in subcellular organelles, including peroxisomes and mitochondria^4^. Although its structure and function remain unknown, TMEM135 may act as a transporter or receptor for metabolites^4^. TMEM135 also influences lipid metabolism, including LD formation and adipogenesis^5,6^. Loss-of-function mutations in TMEM135 prevent hepatic lipid accumulation by enhancing β-oxidation in mice that are obese^7^. TMEM135 depletion enhances lipid accumulation in brown adipose tissue^8^. The effects of TMEM135 on metabolic flexibility and lipid accumulation are inconsistent. Particularly, the role of TMEM135 in intestinal lipid metabolism, including lipid absorption, storage, and utilization, is unknown.

Elongation of very long chain FA protein 6 (ELOVL6), which converts palmitic acid (PA) to stearic acid (SA)^9^, is involved in FA elongation and impacts FA composition of various tissues^10,11^. ELOVL6-deficient mice have increased palmitoleate and decreased stearate levels in the liver^12^. This disruption in hepatic FA composition reduces peroxisome proliferator-activated receptor alpha (PPARα) activation^9^. ELOVL6 negatively affects diabetic and obesity phenotypes^12,13^. Higher ELOVL6 expression worsens non-alcoholic steatohepatitis^13^. Conversely, ELOVL6-deficient mice were protective in diabetic models, experiencing weight loss and improved insulin resistance when fed a high-sucrose and high-fat diet (HFD)^12^. Among ELOVL family, the expression of ELOVL5 and ELOVL6 is higher than any other families for elongation of palmitoleic acid and PA in the intestine. However, no study has reported on the role of intestinal ELOVL6.

PPARα acts as a metabolic sensor, binding to various metabolites such as fibrates and long-chain fatty acids (LCFAs) to activate its function as a transcription factor^14^. Activated PPARα promotes lipid absorption and β-oxidation by targeting genes such as CD36 and acyl-CoA oxidase 1 (ACOX1)^15^. Administration of fibrates, PPARα agonists, enhances β-oxidation and improves lipid uptake^16^. In the aspect of lipid homeostasis, FA uptake and β-oxidation must be tightly regulated to ensure adequate energy production without excess^17^. FAs are oxidized in most tissues to produce energy. Mitochondria are the main organelle involved in FAO, whereas peroxisomes contain a set of enzymes, including ACOX1, that oxidize LCFAs and very LCFAs (VLCFAs) to produce acetyl-CoA^18^. Suppression of β-oxidation-related genes is strongly associated with excessive lipid accumulation and obesity^19,20^. An imbalance in these processes contributes to insulin resistance in obesity and diabetes^17^.

Here, we demonstrate that TMEM135 deficiency contributes to intestinal lipid homeostasis by increasing LCFA uptake and decreasing lipid accumulation through enhanced lipid utilization in mice models fed a HFD or processed lard oil (LO). Our findings highlight the crucial roles of TMEM135 through ELVOL6–OEA–PPARα in lipid uptake and oxidation and clarify their importance in maintaining lipid homeostasis in the intestine.

## Materials and methods

### Animals

TMEM135 knockout (KO) mice were generated at Asan Medical Center (Seoul, Korea) using CRISPR–Cas9 technology. Two single guide RNAs (sgRNAs) were microinjected into fertilized embryos to target TMEM135 (sgRNA1: 5ʹ-TGTACATTACTTTCTTCTGC-3ʹ; sgRNA2: 5ʹ-CCAGTCCGCCTCCTTCCTGA-3ʹ). PCR was performed at 95 °C for 5 min, 95 °C for 30 s, 62.9 °C for 30 s, 72 °C for 30 s (30 cycles), and 72 °C for 5 min. Primer sequences were 5ʹ-GTTAGTCTTTCACACGCGCT-3ʹ and 5ʹ-GTTTCCATTTCAACAAAGCAAATTC-3ʹ. TMEM135^flox/flox^ mice were generated by Cyagen (Jiangsu, China). Exon 2 started ~10.33% of the coding region, and its KO caused a frameshift mutation. Five prime (5ʹ) loxP site was inserted into intron 1 (31,183 bp), and 3ʹ loxP site into intron 2 (2,363 bp). The effective conditional KO (cKO) region was ~628 bp and did not overlap with any other known gene. PCR was performed at 95 °C for 5 min, 95 °C for 30 s, 60 °C for 30 s, 72 °C for 30 s (30 cycles), and 72 °C for 5 min. Primer sequences were 5ʹ-CCTTGATTCCTTAAGCCCACTTG-3ʹ and 5ʹ-GGTTCAGAGATTCAAACAGCAGC-3ʹ. To generate TMEM135 intestinal epithelial-specific cKO (TMEM135^iKO^) mice, we injected 3 × 10^11^ genome copies of AAV9-Alpi-Cre (Vectorbuilder), which had *Alpi* promoter expressed in epithelial cells, by intravenous injection. Transduction efficiency was checked by GFP signal, which was expressed by EGFP control AAV virus (Vectorbuilder). TMEM135 KO efficiency was checked by quantitative PCR analysis. All animal protocols and procedures were approved by the Institutional Animal Care and Use Committee of Gwangju Institute of Science and Technology (Gwangju, Korea). HFD model was generated by feeding 8-week-old mice a 10% kcal fat control normal chow diet (Research Diet, New Brunswick, NJ, USA) or 60% kcal fat diet (HFD, Research Diet) for 22 weeks. For LO challenge, mice were treated with 10 μl/g (body weight) LO after 16 h of starvation. ApoB48 and triglyceride (TG) levels in serum were measured by ApoB48 ELISA kit (MBS028193, MYBioSource) and TG ELISA kit (MBS726589, MYBioSource), respectively. Food intake was measured by metabolic cage analysis from Kyungpook National University Hospital (Daegu, Korea).

### Cell culture and differentiation

Fhs74int (ATCC, Manassas, VA, USA) cells were cultured in Hybri-Care medium (ATCC) supplemented with 90% 30 ng/ml epidermal growth factor and 10% fetal bovine serum (FBS, Gibco) under 5% CO_2_ at 37 °C. Caco-2 cells (ATCC) were cultured in minimum essential medium (MEM) containing 20% FBS, 1× MEM non-essential amino acid solution, 100 μg/ml streptomycin, 100 U/ml penicillin, and 1× GlutaMax (Thermo Fisher Scientific). Cells were differentiated as described previously^21^. Briefly, cells were seeded on Transwell membranes (0.4 μm pore size, Merck) and cultured for 3 weeks until cells adhered via tight junctions. The tight junction was confirmed using transepithelial electrical resistance measurement^21^.

### Isolation and culture of primary intestinal epithelial cells

Cells were isolated as described previously^22,23^. Embryonic day 19 fetuses of mice were used for crypt isolation via cesarean section. The jejunum was isolated and washed with phosphate-buffered saline (PBS, Gibco). The intestine was minced and incubated with type I collagenase and hyaluronidase (Sigma-Aldrich). Cells were centrifuged at 100×*g* for 5 min. Pellets were washed using Dulbecco’s modified Eagle’s medium (DMEM, Gibco) containing 2% FBS and 2% sorbitol. Mixture was centrifuged at 250×*g* for 5 min. Isolated crypts were suspended in DMEM containing 10% FBS. Fibroblasts were removed using 0.25% trypsin (Gibco) after three passages of primary culture. Subsequently, cloning of primary epithelial cells was performed^22^.

### Transfection of siRNA and Myc_TMEM135 plasmids

Small interfering RNAs (Supplementary Table 1) were placed in Opti-MEM (Gibco) with RNAiMAX (Invitrogen) and incubated at room temperature for 30 min before adding to cells. Myc_TMEM135 plasmid was designed and inserted into pcDNA3.1 (Vectorbuilder). Cells were transfected using Lipofectamine 3000 (Thermo Fisher Scientific) with Opti-MEM.

### Isolation of peroxisomes and mitochondria

In total, 2 × 10^8^ cells were detached using trypsin-EDTA. Peroxisomes were isolated using a peroxisome isolation kit (PEROX1, Sigma-Aldrich). Ultracentrifugation (Optima XPN, Beckman Coulter, USA) was performed at 25,000×*g* for 20 min and 100,000×*g* for 1.5 h. Mitochondria were isolated using a mitochondria isolation kit (MITOIS02, Sigma-Aldrich).

### Immunofluorescence staining

Cells were cultured on 12 mm coverslips (Marienfeld, Germany) in 12-well plates, fixed with 4% paraformaldehyde for 20 min at room temperature, and rinsed thrice with 1× PBS. Cells were permeabilized with 0.5% Triton X-100 for 5 min at room temperature, rinsed thrice with 1× PBS, blocked with 3% bovine serum albumin, incubated overnight with primary antibody, and incubated with secondary antibodies (1:500) for 1 h. Nuclei were stained with 4′,6-diamidino-2-phenylindole (1:1,000, Invitrogen). Frozen tissues were sent to OBEN (Suwon, Korea) for slide preparation. Fluorescence was detected using a fluorescence microscope (IX73, Olympus, Japan) and analyzed using the Olympus cellSens Standard software. ImageJ software was used to quantify the fluorescence intensity.

### Lipid extraction and gas chromatography–mass spectrometry (GC–MS)

Lipids were extracted from 3 × 10^6^ cells or 30 mg tissue using the Bligh and Dyer method with FA methyl ester^24^ and diluted using 200 μl of hexane before GC–MS analysis. For GC, injection temperature was set at 230 °C, pressure at 49.7 kPa, total flow at 24.0 ml/min, column flow at 1.00 ml/min, linear velocity at 36.1 cm/s, and purge flow at 3.0 ml/min. For MS, ion source temperature was set at 200 °C and interface temperature at 300 °C.

### Western blotting and fractionation of nucleus, cytosol, and membrane compartments

Cells were harvested from 6 cm dishes and rinsed with 1× PBS containing proteasome inhibitor (GenDEPOT, USA) and phosphatase inhibitors (GenDEPOT). Cells were centrifuged at 4 °C, 13,000 rpm for 15 min and lysed using RIPA buffer. Tissues were rinsed with 1× PBS and homogenized using RIPA buffer. Cells were fractionated using a nucleus/cytosol fractionation kit (Abcam), an ExKine^TM^ membrane, and a cytoplasmic protein extraction kit (Abkine, USA). Equal amounts of proteins were separated via SDS–PAGE and transferred to nitrocellulose membranes. Membranes were blocked with 5% skimmed milk, probed with primary and secondary antibodies (Supplementary Table 2) for 1 h each, and washed with PBS-Tween 20. Protein bands were detected using the ChemiDoc Touch gel imaging system (Bio-Rad, USA).

### Hematoxylin–eosin (H&E), Oil Red O (ORO) staining, and Picrosirius Red staining

The jejunum was isolated and fixed overnight in 4% paraformaldehyde. Tissue H&E staining was performed as described previously^25^. Picrosirius Red staining was performed by OBEN. For ORO staining, tissues were rinsed with 1× PBS, placed into 10% sucrose for 3 h, rinsed again with 1× PBS, transferred to 30% sucrose, and incubated overnight at 4 °C. After fixation, tissues were placed in Cryomold Tissue Tek (Sakura Finetek) with optimal cutting temperature compound (Sakura Finetek). Cryosection was prepared, and ORO staining was performed as described previously^26^.

### RNA-seq and metabolome analysis

For bulk RNA sequencing (RNA-seq) analysis, tissues were harvested and stored in RNAlater (Thermo Fisher Scientific) at 4 °C. Transcriptome sequencing was performed using the Theragen Bio software (Seoungnam, Korea). FASTaq files were analyzed using RNASTAR and DESeq2 in R. For metabolomic analysis, feces was collected and sent to Metabolon Inc. (Durham, NC, USA). RNA-seq data of patient with Crohn’s disease (CD) were obtained from Gene Expression Omnibus (GEO), which were supported from previous report (GSE93624)^27^. Samples were divided equally and used for liquid chromatography/tandem mass spectrometry (LC/MS/MS). Proprietary software was used to match ions to an in-house library of standards to identify the metabolites. Metabolite quantification was performed using peak area integration. The obtained data were normalized by volume and analyzed. Statistical analyses were performed using the log-transformed data. Volcano plot from metabolomics analysis was drawn using MetaboAnalyst^28^.

### Seahorse analysis

Cells and isolated peroxisomes were seeded in XFe96 cell culture microplates (Agilent, USA). Before analysis, XFe96 sensor cartridge (Agilent) was soaked in Seahorse XF calibrant solution (Agilent) and incubated at 37 °C in a non-CO_2_ incubator. DMEM was replaced with Seahorse XF DMEM (pH 7.4, Agilent) supplemented with 1 mM pyruvate, 10 mM glucose, and 2 mM glycine, and culture microplates were incubated for 1 h at 37 °C non-CO_2_ incubator. Seahorse analysis was then conducted according to the instructions of a Seahorse XF Cell Mito stress test kit (Agilent) using a Seahorse XFe96 analyzer (Agilent).

### Quantitative reverse transcription–PCR (qRT–PCR)

Cells and homogenized tissues were placed in TRIzol (Thermo Fisher Scientific) to extract RNA. RNAs were used to synthesize cDNA using the Transcriptor first strand cDNA synthesis kit (Roche, Switzerland). cDNAs were used as templates for qRT–PCR using primers (Supplementary Table 3) and FastStart DNA Master SYBR Green (Roche). mRNA expression was analyzed using CFX Connect real-time PCR detection system (Bio-Rad).

### Free fatty acid (FFA) uptake assay

FFA uptake kit (Abcam) and BODIPY-Palmitate (Cayman, Ann Arbor, MI, USA) were used. Fluorescence was measured using a fluorometer (CLARIOstar^Plus^; BMG LABTECH, Germany).

### Stable isotope analysis of fatty acids

Cells were cultured with U-¹³C₁₆-palmitate (CLM-409, Cambridge Isotope Laboratories, USA) for the indicated times and washed twice with ice-cold PBS before harvest. Total lipids were extracted using a modified Bligh and Dyer method^24^. The organic phase was collected, dried under nitrogen, and reconstituted in isopropanol/acetonitrile (9:1) before analysis. Lipid extracts were separated on a column using mobile phase A consisting of acetonitrile/water (60:40) and mobile phase B consisting of isopropanol/acetonitrile (90:10), each supplemented with 10 mM ammonium formate and 0.1% formic acid. Mass spectrometric analysis was performed in positive and negative electrospray ionization modes using high-resolution full-scan acquisition. Incorporation of U-¹³C₁₆-palmitate into lipid species was determined based on isotopolog mass shifts after correction for natural isotope abundance. FA uptake rate was expressed as the labeling velocity. Labeling velocity was approximated as the slope of U-^13^C_16_-palmitate incorporation over time: labeling velocity (%/h) = Δ labeled fraction (%)/Δ time (h). For two-point comparisons, labeling velocity was calculated as: labeling velocity (%/h) = (labeled fraction at *t*₂ − labeled fraction at *t*₁)/(*t*₂ − *t*₁), in which *t*₁ and *t*₂ correspond to the tracer incubation time points. Three independent biological replicates were analyzed for each condition.

### PPRE-luciferase activity assay

PPRE X3-TK-luc was a gift from Bruce Spiegelman (Addgene, USA)^29^. PPAR transcriptional activity was assessed using a dual-luciferase reporter assay. Cells were seeded in culture plates and transiently co-transfected with the PPRE-driven firefly luciferase reporter plasmid together with the Renilla luciferase control vector pRL-TK (E2241, Promega, USA) using Lipofectamine 3000 (Thermo Fisher Scientific), according to the manufacturer’s instructions. After transfection, cells were incubated for 24 h and then treated under the indicated experimental conditions. Luciferase activity was measured using the Firefly/Renilla Dual Luciferase Assay Kit (SCT152, MilliporeSigma, USA). Firefly luciferase activity was normalized to Renilla luciferase activity to correct for transfection efficiency. Luminescence signals were detected using a CLARIOstar Plus microplate reader (BMG LABTECH, Germany).

### TMEM135 structure analysis

TMEM135 structure modeling was performed by CALICI (Daejeon, Korea). TMEM135 structural modeling and molecular docking were performed using the internal molecular docking module implemented in Pharmaco-net. The 3D structure of TMEM135 was prepared and optimized before docking analysis, and docosahexaenoic acid (DHA) was used as the ligand for interaction simulation. To reproduce the native membrane-associated environment of TMEM135, membrane system modeling was generated using CHARMM-GUI Membrane Builder with a biologically relevant lipid bilayer composition. The membrane-embedded TMEM135 model was structurally refined before docking calculations. Molecular docking was then conducted to predict the binding mode and affinity of DHA toward TMEM135 under default Pharmaco-net docking parameters unless otherwise specified. Visualization of docking poses, binding pocket identification, and residue-level interaction analysis were performed using PyMOL (Schrödinger, LLC). The final docking conformations were selected based on the predicted binding affinity score and consistency with the transmembrane topology of TMEM135.

### Intestinal permeability test

Mice were orally gavaged with fluorescein isothiocyanate–dextran (FITC-dextran, 4 kDa; 46944, Sigma-Aldrich) at a dose of 600 mg/kg body weight. Blood sample was collected 4 h after gavage, and serum was isolated by centrifugation. Serum fluorescence was measured using a CLARIOstar Plus microplate reader (BMG LABTECH, Germany) at excitation andemission wavelengths of 490/520 nm to assess intestinal permeability.

### Reagents

ELOVL6-IN-1, ELOVL6-IN-2, LEI-401, CAY10566, OEA, sulfo-*N*-succinimidyl oleate, GW6471, and trichosadiynoic acid (TDYA) were purchased from MedChemExpress (MCE, USA). SIRT1 activity and NAD^+^/NADH ratio were measured using a SIRT1 Activity Assay kit (ab156065, Abcam) and an NAD^+^/NADH Assay kit (ab65348, Abcam), respectively.

### Statistical analysis

All statistical analyses were performed by unpaired two-tailed Student’s *t* tests. Two-way analysis of variance (ANOVA) was used to determine significance among more than two groups followed by Tukey’s post hoc tests. Differences were considered statistically significant at *P* < 0.05. Data visualizations were created using Prism 9.

## Results

### LCFA contents are decreased in the intestine of HFD-fed TMEM135 KO mice

**Fig. 1:**
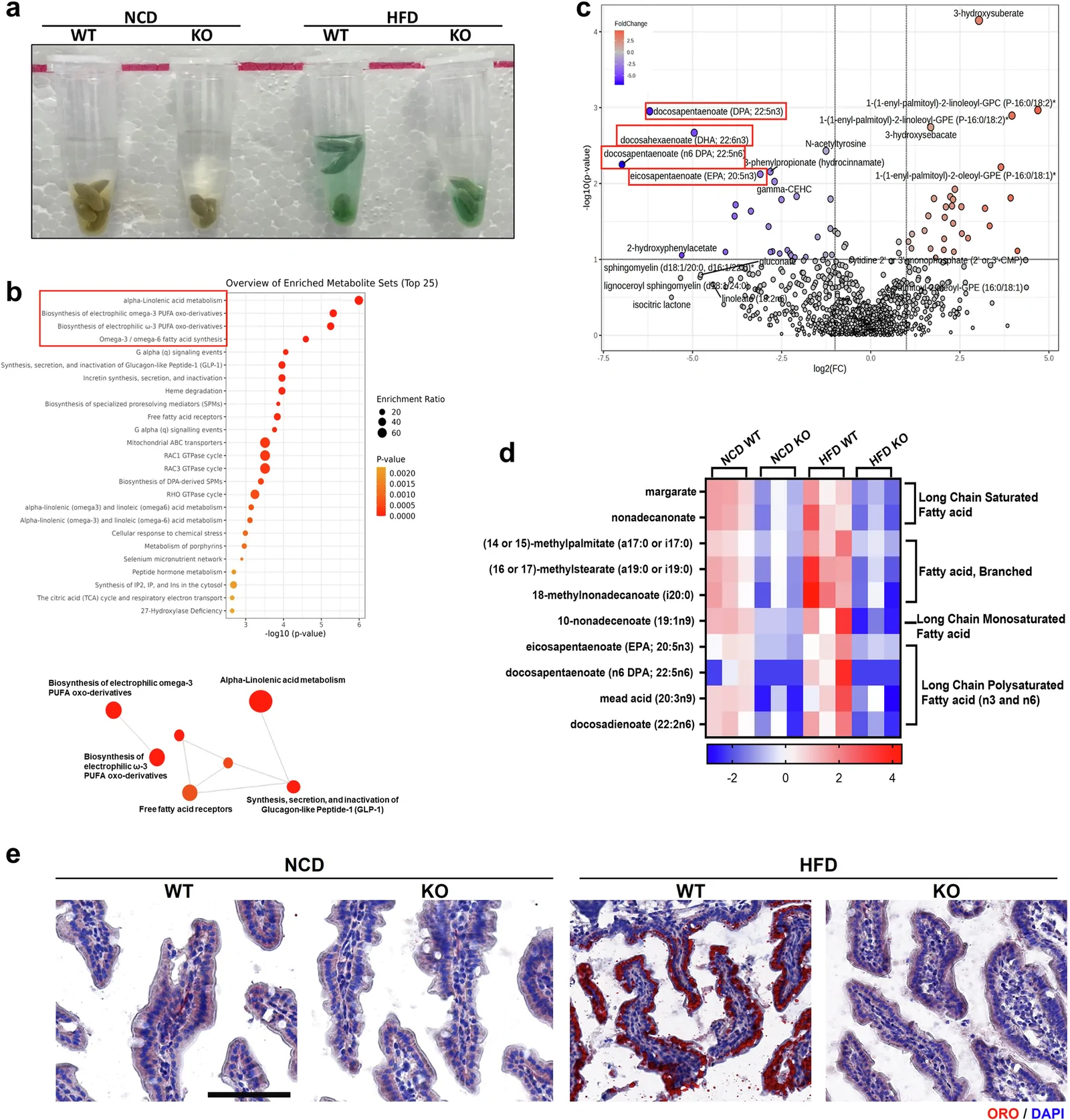
Difference in long-chain fatty acids composition between WT and TMEM135 KO mice. **a**–**e** Mice were fed either NCD or HFD for 22 weeks (*n* = 3 per group). **a** Feces floated on water. **b**–**d** Metabolites from feces were analyzed by metabolomics. Gene set enrichment analysis, and bubble plot (part **b**) volcano plot (part **c**) and heat map (part **d**). **e** Oil O Red staining of the jejunum. Scale bar, 50 μm. DAPI 4′,6-diamidino-2-phenylindole, FC fold change, HFD high-fat diet, KO knockout, NCD normal chow diet, PUFA polyunsaturated fatty acid, WT wild-type.

To investigate whether TMEM135 has functional significance in intestinal lipid handling, we next examined the physiological consequences of TMEM135 deficiency in vivo using a mouse model. Wild-type (WT) and TMEM135 KO mice were fed a HFD. Feces from TMEM135 KO mice fed a HFD sank and did not float in water (Fig. 1a). This led to the hypothesis that feces from TMEM135 KO mice contained fewer lipids than WT mice. Gene set enrichment analysis (GSEA) of metabolomics showed several metabolite sets related to LCFAs, including alpha-linolenic acid and polyunsaturated fatty acids, which were significantly changed in TMEM135 KO mice feces compared with WT mice (Fig. 1b).

Various LCFA levels were reduced in TMEM135 KO mice feces compared with WT mice regardless of diet type (Fig. 1c). Moreover, although many LCFAs were increased in HFD-fed WT mice compared with normal chow diet-fed WT mice, no increase was observed in TMEM135 KO mice (Fig. 1d). Consistently, intestinal epithelial cells from HFD-fed WT mice had increased lipid accumulation as indicated by ORO staining, whereas ORO intensity in TMEM135 KO mice remained unchanged regardless of diet (Fig. 1e).

### TMEM135 depletion enhances lipid absorption during the early stage of LO treatment

**Fig. 2:**
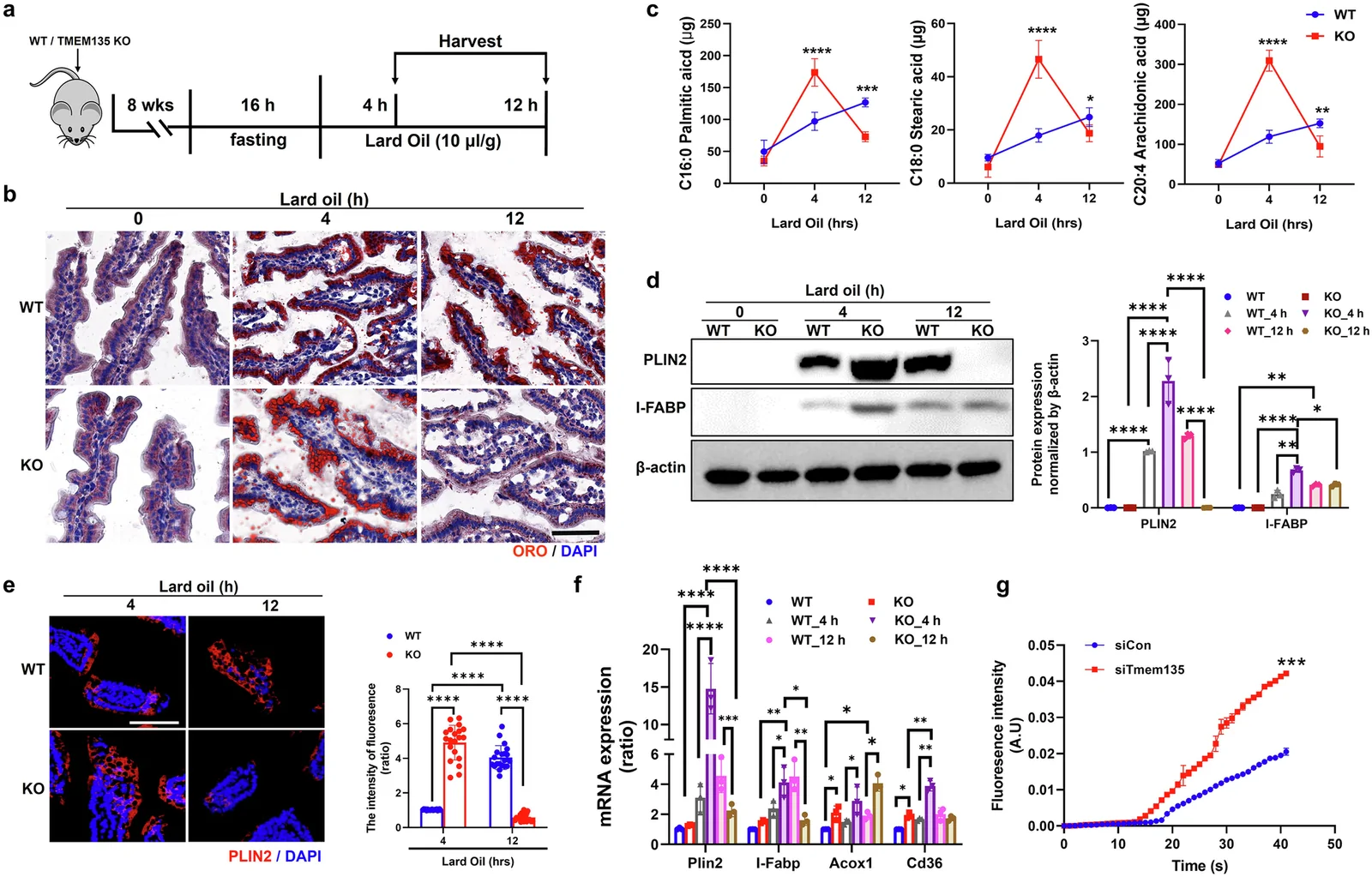
Rapid uptake of long-chain fatty acids in the intestine of TMEM135 KO mice after lard oil treatment. **a**–**f** Mice were orally administered with lard oil (10 μl/g body weight, *n* = 3 per group). **a** Experimental scheme. **b** ORO staining of the jejunum. Scale bar, 50 μm. **c** Gas chromatography–mass spectrometry analysis of palmitic acid (C16:0), stearic acid (C18:0), and arachidonic acid (C20:4). **d** Western blot and quantification for PLIN2, I-FABP, and β-actin. **e** Immunofluorescence staining and quantification for PLIN2. Scale bar, 50 μm. **f** mRNA expression of *Plin2*, *I-Fabp*, and *Acox1*. **g** BODIPY-Palmitate fluorescence in Fhs74int cells transfected with siCon and siTMEM135. *n* = 3 independent experiments. All data are represented as mean ± SD, *P < 0.05, **P < 0.01, ***P < 0.001, ****P < 0.0001. A.U. arbitrary unit, DAPI 4′,6-diamidino-2-phenylindole, I-FABP intestinal fatty acid-binding protein, KO knockout, ORO Oil Red O, TMEM transmembrane, WT wild-type.

To investigate the differences between WT and TMEM135 KO mice, lipid contents were assessed after LO administration (Fig. 2a). In intestinal epithelial cells of WT mice, ORO intensity increased in a time-dependent manner. However, in TMEM135 KO mice, ORO intensity increased 4 h after LO treatment and then decreased (Fig. 2b). LCFA contents, including PA (C16:0), SA (C18:0), and arachidonic acid (C20:4, AA), increased in a time-dependent manner after LO treatment in WT mice. In TMEM135 KO mice, LCFA levels increased 4 h after LO treatment and then decreased after 12 h (Fig. 2c). Consistently, PLIN2 expression in WT intestine increased in a time-dependent manner by LO treatment. However, in TMEM135 KO mice, PLIN2 expression was induced 4 h after LO treatment and then disappeared (Fig. 2d–f). Notably, 4 h after LO treatment, PLIN2 expression in TMEM135 KO mice was markedly higher than that in WT mice. Meanwhile, the expression of intestinal FA-binding protein, which binds to absorbed FA for delivery to specific suborganelles in the intestine^30^, was also increased in TMEM135 KO mice compared with WT mice 4 h after LO treatment. However, intestinal FA-binding protein expression was decreased after 12 h in TMEM135 KO mice intestine (Fig. 2d–f). Moreover, siTMEM135-transfected Fhs74int cells (Supplementary Fig. 1a) exhibited a rapid increase in BODIPY-Palmitate uptake than siCon cells in a time-dependent manner (Fig. 2g). These results suggest that TMEM135 deficiency increases lipid absorption without inducing intestinal lipid accumulation.

To elucidate the physiological consequences of intestinal TMEM135 deficiency, we evaluated indicators of inflammation and tissue remodeling. Specifically, validation of the expression of pro-inflammatory genes, including *Tnfa* and *Il-6*, revealed no distinct differences between WT and TMEM135-deficient mice, either under basal conditions or following 4 h of LO treatment (Supplementary Fig. 1b). Furthermore, histological analysis using Picrosirius Red staining showed no detectable increase in collagen deposition, demonstrating the absence of fibrosis (Supplementary Fig. 1c). Taken together, these results suggest that TMEM135 deficiency does not induce overt inflammation or fibrotic remodeling in the intestine under the conditions tested in this study, without any of impairment in intestinal integrity.

### Lipid uptake is enhanced by CD36 in TMEM135-deficient intestine

**Fig. 3:**
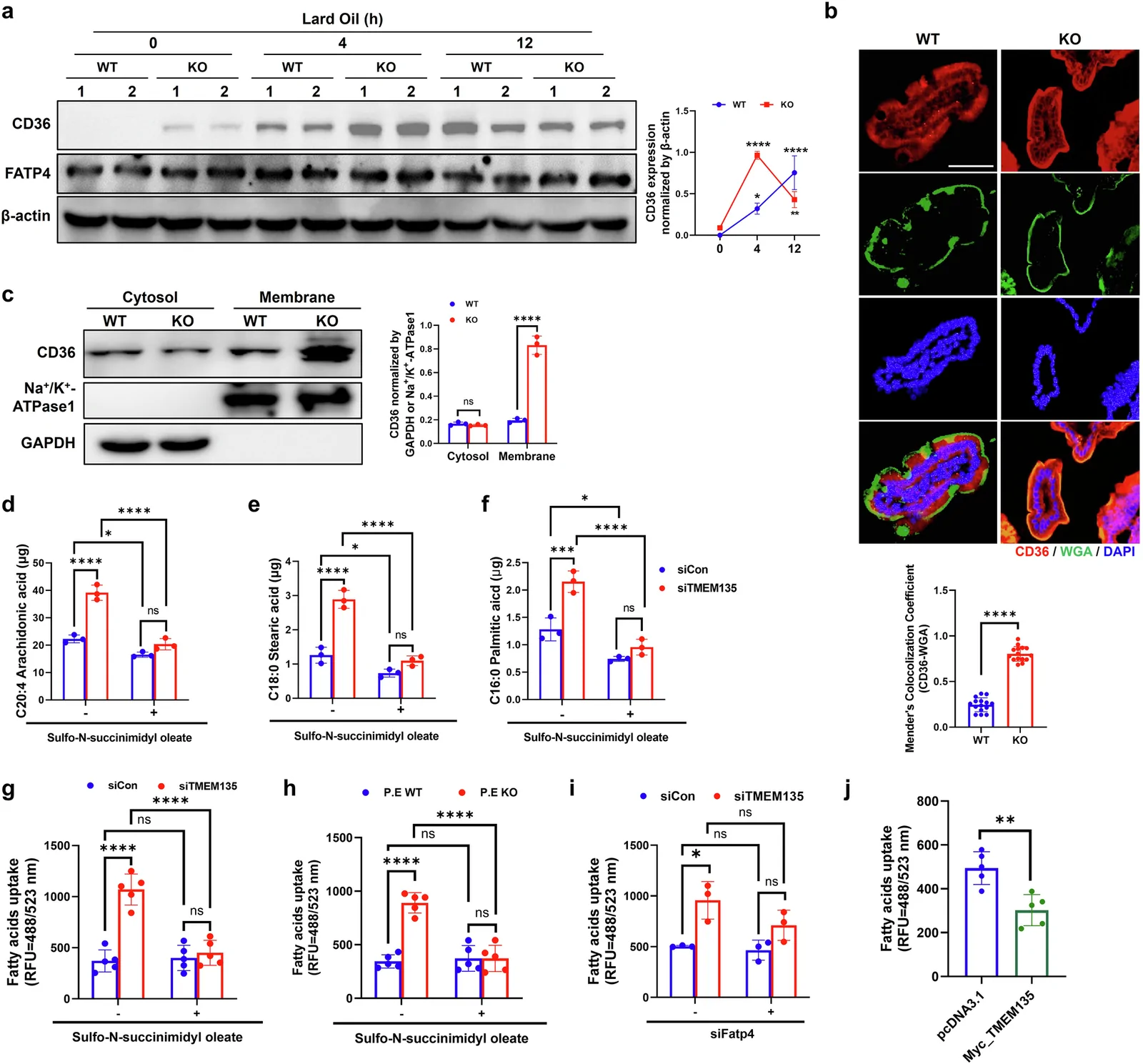
CD36 is a major lipid uptake receptor in TMEM135-depleted intestines after lard oil treatment. **a**–**c** Mice were orally administrated with lard oil (10 μl/g body weight, *n* = 3 per group). **a** Western blot and quantification for CD36 and β-actin. **b** Immunofluorescence staining of CD36 and WGA, and Menders’ colocalization coefficient. Scale bar, 50 μm. **c** Membrane fractionation, western blot, and quantification for CD36, Na^+^/K^+^-ATPase1, and GAPDH. **d**–**f** Fhs74Int cells were transfected with siCon or siTMEM135 for 12 h and treated with sulfo-*N*-succinimidyl oleate (50 μM) for 6 h. *n* = 3 independent experiments. Gas chromatography–mass spectrometry analysis of palmitic acid (C16:0) (part **d**) stearic acid (C18:0) (part **e**) and arachidonic acid (C20:4) (part **f**). **g**–**j** Fatty acid uptake assay in each condition. *n* = 5 independent experiments. **g** Fhs74Int cells were transfected with siCon or siTMEM135 for 12 h and treated with sulfo-*N*-succinimidyl oleate (50 μM) for 6 h. **h** The isolated primary epithelial cells from the intestine of WT (PE WT) and TMEM135 KO (PE KO) mice treated with 50 μM sulfo-*N*-succinimidyl oleate. **i** Fhs74Int cells transfected with siFATP4 for 6 h after siCon or siTMEM135 transfection for 12 h. **j** Fhs74int cells transfected with Myc_TMEM135 for 12 h. All data are presented as mean ± SD, *P < 0.05, **P < 0.01, ***P < 0.001, ****P < 0.0001. FATP4 fatty acid transport protein 4, KO knockout, ns not significant, RFU related fluorescence unit, WT wild-type.

CD36 is a well-established mediator of LCFA uptake in intestinal epithelial cells and has been described as a key facilitator of lipid absorption in the small intestine^31,32^. In addition, we confirmed that *Cd36* expression was upregulated in TMEM135 KO intestine (Fig. 2f). Therefore, we investigated how CD36 expression changes under TMEM135-deficient conditions to determine whether the increased FD uptake could be explained, at least in part, by changes in this key transporter. CD36 expression was increased in a time-dependent manner in WT mice following LO treatment. However, in TMEM135 KO mice, CD36 expression peaked at 4 h after LO treatment and subsequently decreased (Fig. 3a). Particularly, CD36 expression was higher on the membrane of epithelial cells in TMEM135 KO mice compared with WT mice in 4 h LO treatment (Fig. 3b,c). Meanwhile, the expression of another lipid transporter, FA transport protein 4 (FATP4), was unchanged in the intestine of both mice treated with LO (Fig. 3a).

Next, to confirm FA uptake by CD36, cells were treated with sulfo-*N*-succinimidyl oleate, a CD36 inhibitor. The concentrations of PA, SA, and AA were increased in siTMEM135 cells compared with siCon cells. However, sulfo-*N*-succinimidyl oleate treatment significantly reduced all LCFA levels in both cells (Fig. 3d–f). Additionally, related fluorescence unit (RFU) analysis revealed that siTMEM135 cells increased FA uptake compared with control cells, which was also inhibited by sulfo-*N*-succinimidyl oleate (Fig. 3g). A similar effect was observed in isolated primary epithelial cells (Fig. 3h). Consistent with no changes in the FATP4 expression level (Fig. 3a), depletion of FATP4 by siFATP4 did not affect lipid uptake in siTMEM135 cells (Fig. 3i). By contrast, TMEM135 overexpression reduced FA uptake in Fhs74int cells compared with control cells (Fig. 3j). These data suggest that CD36 is the main transporter contributing to increased FA uptake upon TMEM135 depletion.

### PPARα activation regulates lipid uptake under TMEM135 depletion

Among several transcriptional factors^15^ that regulate CD36 expression, we observed that PPARα target gene expression was upregulated, whereas target genes of other transcriptional factors, such as PPARγ, STAT3, HIF1-α, and NF-κB, were unchanged (Supplementary Fig. 1d). In WT mice, PPARα was markedly observable in the cytosolic fraction, whereas PPARα was expressed in both the cytosolic and nuclear fractions in TMEM135 KO mice intestine (Supplementary Fig. 1e). Consistently, siTMEM135 transfection induced a time-dependent nuclear translocation of PPARα (Supplementary Fig. 1f). Although *Ppar*α expression was not changed, mRNA expression of target genes, such as *Cd36*, *Acox1*, and *Abcd3*, was increased upon TMEM135 depletion. However, treatment with GW6471, a PPARα antagonist, significantly suppressed the upregulation of PPARα target genes in siTMEM135 cells (Supplementary Fig. 1g).

**Fig. 4:**
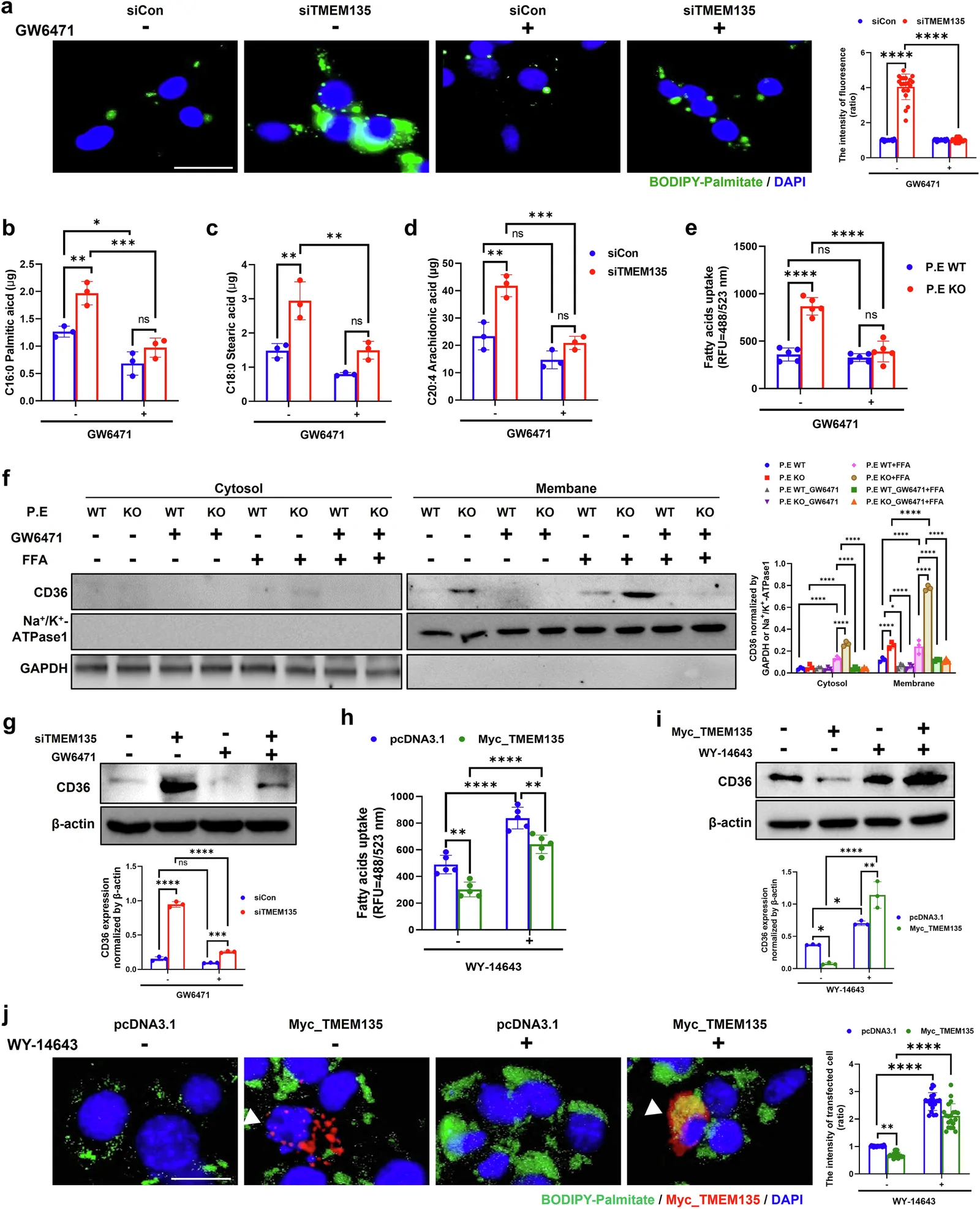
Effect of PPARα on fatty acid uptake under TMEM135 depletion. **a**–**d** Fhs74Int cells were treated with GW6471 (5 μM) for 1 h and transfected with siCon or siTMEM135 for 12 h. **a** BODIPY-Palmitate fluorescence and quantification. Scale bar, 10 μm. Gas chromatography–mass spectrometry analysis of palmitic acid (C16:0) (part **b**) stearic acid (C18:0) (part **c**) and arachidonic acid (C20:4) (part **d**) *n* = 3 independent experiments. **e** Fatty acid uptake assay of isolated primary epithelial cells from the intestine of WT and TMEM135 KO mice treated with GW6471 (5 μM) for 1 h, *n* = 5 independent experiments. **f** Primary epithelial cells isolated from the intestine of WT and TMEM135 KO mice were treated with GW6471 (5 μM) for 1 h, followed by treatment with or without FFA (200 μM). Membrane fractionation, western blot, and quantification for CD36 and Na^+^/K^+^-ATPase1 (membrane) or GAPDH (cytosol), *n* = 3 independent experiments. **g** Western blot and quantification for CD36 and β-actin in Fhs74Int cells transfected with siCon or siTMEM135 for 12 h after GW6471 (5 μM) treatment for 1 h. *n* = 3 independent experiments. **h**–**j** Fhs74Int cells transfected with pcDNA3.1 or Myc_TMEM135 for 12 h after treatment with WY-14643 (25 μM) for 1 h. *n* = 5 independent experiments. **h** Fatty acid uptake assay in each condition. *n* = 5 independent experiments. **i** Western blot and quantification for CD36 and β-actin. *n* = 3 independent experiments. **j** BODIPY-Palmitate fluorescence and quantification. Scale bar, 5 μm. All data represent mean ± SD, *P < 0.05, **P < 0.01, ***P < 0.001, ****P < 0.0001. DAPI 4′,6-diamidino-2-phenylindole, FFA free fatty acid, KO knockout, ns not significant, PE primary epithelial, RFU related fluorescence unit, TMEM135 transmembrane protein 135, WT wild-type.

Next, we examined the effect of PPARα on lipid uptake. GW6471 treatment inhibited BODIPY-Palmitate uptake and increased lipid content in siTMEM135 cells (Fig. 4a–d). Furthermore, the increased FA uptake induced by TMEM135 depletion was reduced by GW6471 in primary epithelial cells from TMEM135 KO mice and siTMEM135 cells (Fig. 4e and Supplementary Fig. 2a). Similar results were also observed in FFA-treated cells, as shown by ORO staining (Supplementary Fig. 2b). In siTMEM135 cells, GW6471 treatment reduced the increased CD36 localization in the membrane and cytoplasm (Supplementary Fig. 2c).

To confirm that PPARα regulates FA uptake through CD36, we investigated whether FFAs affected the temporal changes and membrane recruitment of CD36 under TMEM135 depletion. CD36 expression was higher in TMEM135 KO epithelial cells and was more plasma membrane-localized compared with WT cells without FFA treatment (Fig. 4f). After FFA treatment, both temporal expression and membrane translocation of CD36 remained higher in TMEM135 KO than in WT cells, and these changes were increased compared to before FFA treatment (Fig. 4f and Supplementary Fig. 2d). Additionally, FFA treatment increased cytosolic localization of CD36 in TMEM135 KO cells, which was not evident before FFA exposure (Fig. 4f). GW6471 treatment reduced CD36 expression and its localization on the plasma membrane and cytosol in TMEM135 KO cells (Fig. 4f and Supplementary Fig. 2d). Moreover, GW6471 treatment markedly reduced CD36 upregulation in siTMEM135 cells (Fig. 4g). DMSO, used as a vehicle control at the same concentration applied for GW6471, did not affect CD36 localization (Supplementary Fig. 2e). By contrast, FA uptake was decreased in Myc_TMEM135-transfected cells but, a PPARα agonist, WY-14643 treatment increased FA uptake in both cells, although Myc_TMEM135 cells were lower than control cells (Fig. 4h). Consistently, TMEM135 overexpression reduced CD36 expression, whereas WY-14643 treatment reversed CD36 expression (Fig. 4i). Furthermore, Myc_TMEM135 cells showed lower BODIPY-Palmitate intensity compared with non-transfected cells (Fig. 4j). However, WY-14643 treatment increased BODIPY-Palmitate uptake in Myc_TMEM135 cells. The transfection efficiency and effects of WY-14643 were confirmed by mRNA levels of TMEM135 and PPARα target genes (Supplementary Fig. 2f). These results demonstrate that CD36-mediated lipid uptake is enhanced by PPARα activation under TMEM135 depletion.

### PPARα is activated by ELOVL6 under TMEM135 depletion

**Fig. 5:**
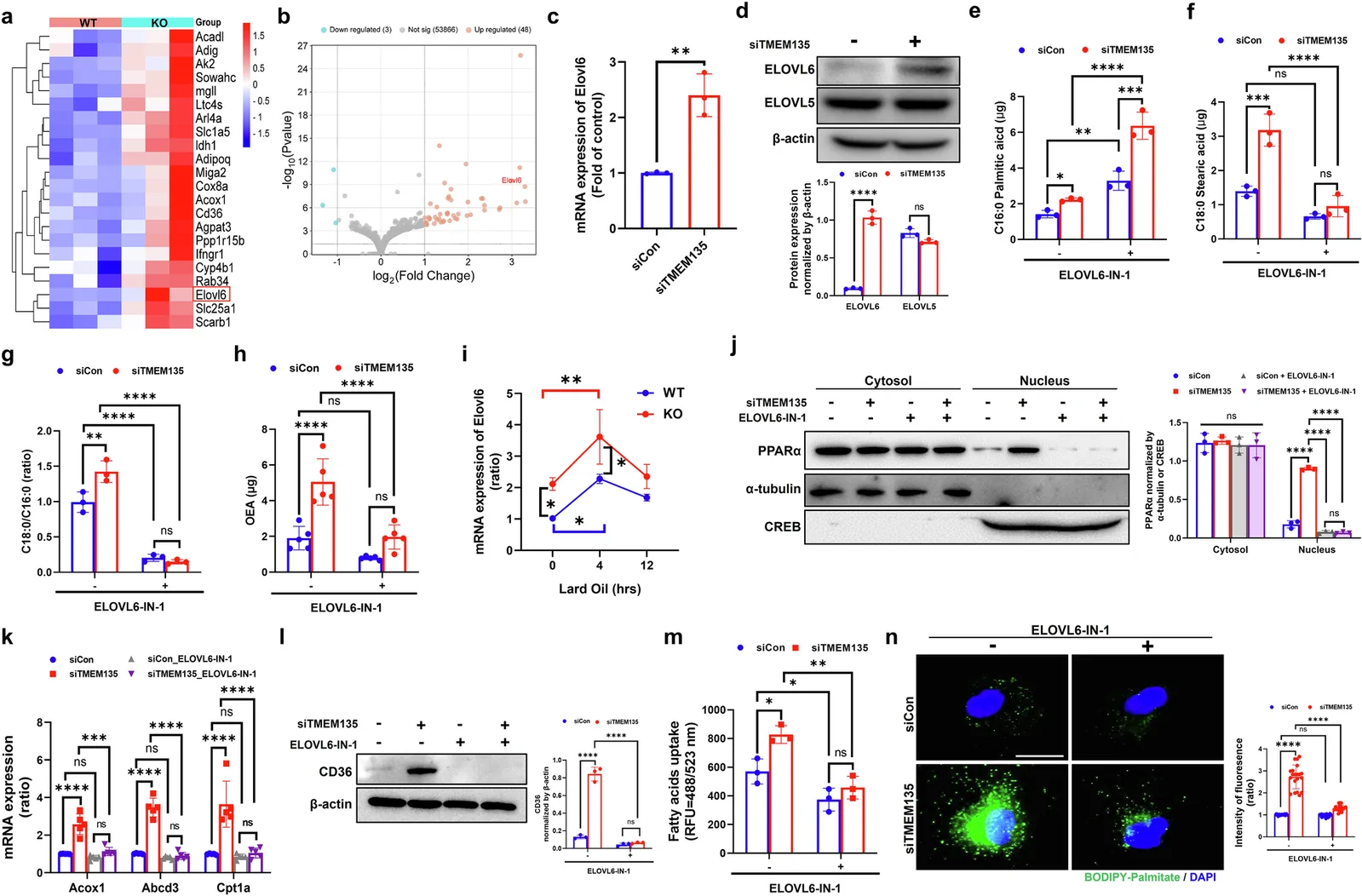
Effect of ELOVL6 on lipid contents under TMEM135 depletion. **a**,**b** Bulk RNA-seq analysis from the intestine of WT and TMEM135 KO mice (*n* = 3). **a** Heatmap. **b** Volcano plot. **c**,**d** Fhs74Int cells transfected with siCon or siTMEM135 for 12 h. **c** *Elovl6* mRNA expression, *n* = 3 independent experiments. **d** Western blot and quantification for ELOVL6, ELOVL5, and β-actin, *n* = 3 independent experiments. **e**–**h** Transfected cells treated with an ELOVL6 inhibitor. Gas chromatography–mass spectrometry analysis of palmitic acid (C16:0) (part **e**) stearic acid (C18:0) (part **f**) C18:0/C16:0 ratio (*n* = 3) (part **g**) and OEA (*n* = 5) (part **h**). **i** *Elovl6* mRNA expression in the jejunum of WT and TMEM135 KO mice after lard oil administration (10 μl/g body weight, *n* = 3 per group). **j**–**n** Fhs74int cells were treated with ELOVL6-IN-1 (10 μM) for 1 h and transfected with siCon or siTMEM135 for 12 h. **j** Nucleus fractionation, western blot, and quantification for PPARα and α-tubulin (cytosol) or CREB (nuclear fraction), *n* = 3 independent experiments. **k** mRNA expression, *n* = 5 independent experiments. **l** Western blot and quantification for CD36 and β-actin, *n* = 3 independent experiments. **m** Fatty acid uptake assay, *n* = 3 independent experiments. **n** BODIPY-Palmitate fluorescence and quantification. Scale bar, 5 μm. All data represent mean ± SD, *P < 0.05, **P < 0.01, ***P < 0.001, ****P < 0.0001. DAPI 4′,6-diamidino-2-phenylindole, KO knockout, ns not significant, OEA oleoylethanolamide, PPARα peroxisome proliferator-activated receptor alpha, RFU related fluorescence unit, WT wild-type.

RNA-seq analysis identified several genes that were upregulated in TMEM13 KO mice intestine compared with WT. *Elovl6* was significantly upregulated in TMEM135 KO mice (Fig. 5a,b). ELOVL6 mRNA and protein expression were also increased in siTMEM135 cells (Fig. 5c,d). As ELOVL6 expression was induced, its activity was also increased in TMEM135 depletion. SA concentration was markedly increased, and C18:0/16:0, which indicated the activity of ELOVL6, was elevated in siTMEM135 cells (Fig. 5e,f). However, ELOVL6-IN-1, an ELOVL6 inhibitor, treatment increased PA and decreased SA content in both cells (Fig. 5e,f). The increased C18:0/16:0 ratio was also reduced by ELOVL6-IN-1 (Fig. 5g). Furthermore, the increase in oleoylethanolamide (OEA), a downstream metabolite of ELVOL6 induced by TMEM135 depletion, was significantly reduced by ELOVL6-IN-1 treatment (Fig. 5h). In addition, *Elovl6* expression in the intestines, including jejunum and duodenum, of TMEM135 KO mice was higher at 0 h and 4 h after LO challenge compared with WT mice. However, no significant difference was observed at 12 h (Fig. 5i and Supplementary Fig. 3a). These results suggest that TMEM135 may be involved in ELOVL6 regulation during early lipid challenge in the intestines. Next, we investigated the relationship between ELOVL6 and PPARα. First, we examined the effect of ELOVL6 inhibition on PPARα activation. ELOVL6-IN-1 treatment reduced nuclear translocation of PPARα and increased mRNA expression of PPARα target genes, including *Acox1*, *Abcd3*, and *Cpt1a*, in TMEM135-deficient cells (Fig. 5j,k). Furthermore, the increased CD36 expression caused by TMEM135 depletion was reduced by ELOVL6 inhibition (Fig. 5l). The increased FA and BODIPY-Palmitate uptake in siTMEM135 cells was also reduced by ELOVL6-IN-1 treatment (Fig. 5m,n).

**Fig. 6:**
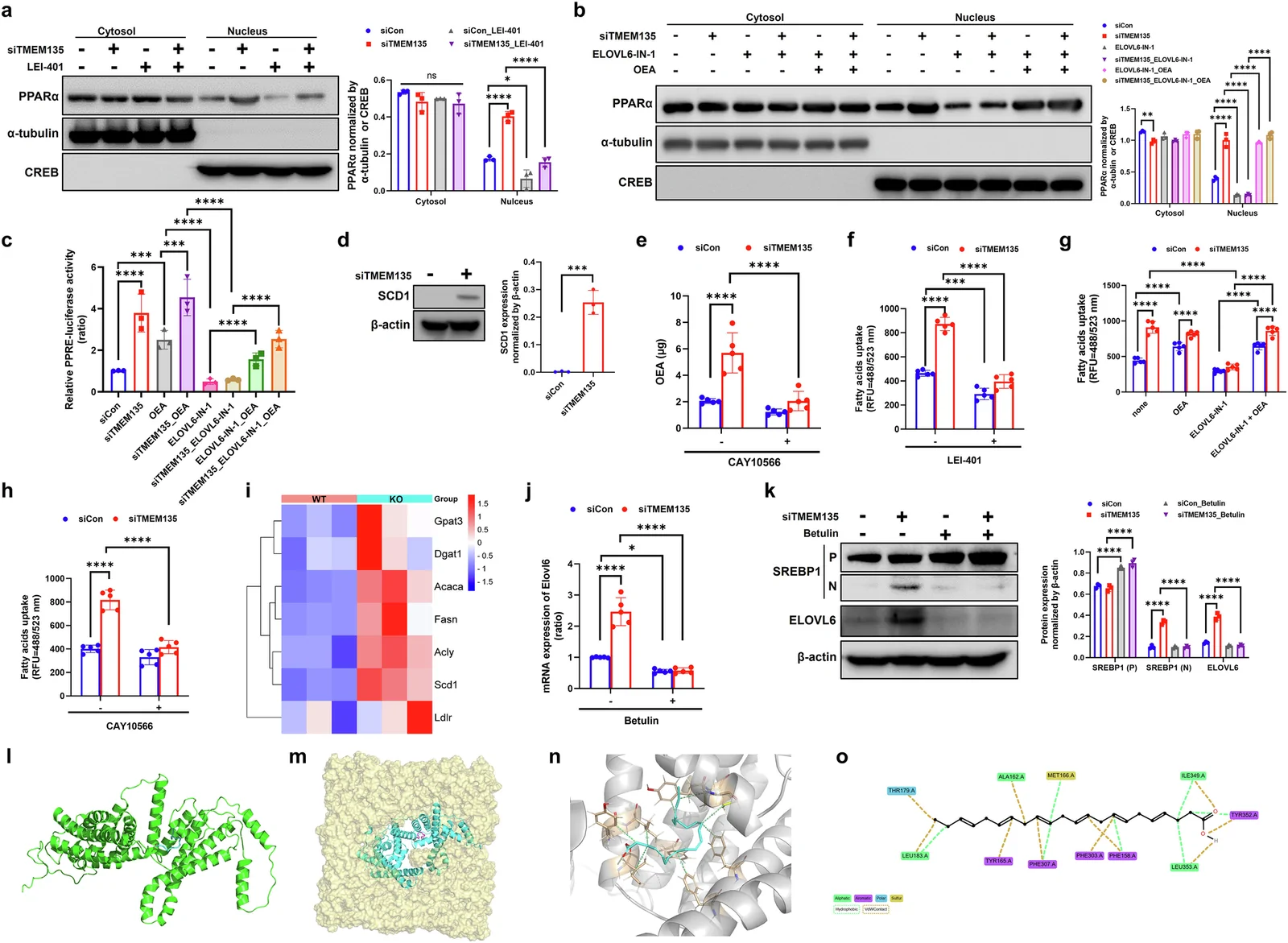
Effect of OEA via ELOVL6 on PPARα activation under TMEM135 depletion. **a** Fhs74Int cells were treated with LEI-401 (10 μM) for 1 h and transfected with siCon or siTMEM135 for 12 h. Nucleus fractionation, western blot, and quantification for PPARα and α-tubulin (cytosol) or CREB (nuclear fraction), *n* = 3 independent experiments. **b**,**c** Fhs74int cells were treated with ELOVL6-IN-1 (10 μM) for 1 h and transfected with siCon or siTMEM135 for 12 h. Transfected cells were treated with OEA (10 μM). **b** Nucleus fractionation, western blot, and quantification for PPARα and α-tubulin (cytosol) or CREB (nuclear fraction), *n* = 3 independent experiments. **c** PPRE-luciferase activity was measured, *n* = 3 independent experiments. **d** Western blot and quantification for SCD1 and β-actin in Fhs74Int cells transfected with siCon or siTMEM135 for 12 h. *n* = 3 independent experiments. **e** Gas chromatography–mass spectrometry analysis of OEA (*n* = 5).Fatty acid uptake assay of Fhs74int cells treated with LEI-401 (10 μM) for 1 h (part **f**) ELOVL6-IN-1 (10 μM) for 1 h before OEA (10 μM) treatment (part **g**) or CAY10566 (5 μM) for 1 h and transfected with siCon or siTMEM135 for 12 h (part **h**). *n* = 5 independent experiments. **i** Heatmap, RNA-seq analysis from the intestine of WT and TMEM135 KO (*n* = 3). **j**,**k** Fhs74Int cells were treated with betulin (50 μM) for 1 h and transfected with siCon or siTMEM135 for 12 h. **j** *Elovl6* mRNA expression, *n* = 5. **k** Western blot and quantification for SREBP1, ELOVL6, and β-actin. P and N represented before and after proteolytic cleavage of SREBP1, respectively. *n* = 3 independent experiments. **l**–**o** Structural modeling of TMEM135 and predicted DHA-binding pocket. **l** Predicted 3D structure of TMEM135 protein. **m** TMEM135 embedded in a membrane model generated using CHARMM-GUI, showing its transmembrane localization. **n** Enlarged view of the predicted DHA-binding pocket within TMEM135, highlighting the docking pose of DHA in the binding cavity. **o** Two-dimensional interaction map of DHA with amino acid residues in the TMEM135 binding site, indicating hydrophobic interactions and hydrogen bonding. All data represent mean ± SD, *P < 0.05, **P < 0.01, ***P < 0.001, ****P < 0.0001. KO knockout, OEA oleoylethanolamide, PPARα peroxisome proliferator-activated receptor alpha, RFU related fluorescence unit, SCD1 stearoyl-CoA desaturase 1, TMEM135 transmembrane protein 135, vdW van der Waals, WT wild-type.

Next, we further accessed the effect of OEA on PPARα activation through ELOVL6. *N*-acyl-phosphatidylethanolamine (NAPE)-PLD catalyzes the conversion of NAPE into *N*-acylethanolamines, such as arachidonoylethanolamide and OEA^33^. LEI-401, a NAPE-PLD inhibitor, treatment reduced nuclear translocation of PPARα in both siCon and siTMEM135 cells, with a more pronounced effect observed in siTMEM135 cells (Fig. 6a). As shown in the previous results, both OEA level and PPARα nuclear translocation induced by siTMEM135 were attenuated by ELOVL6-IN-1 treatment (Figs. 5j and 6b). Additionally, exogenous OEA treatment restored PPARα nuclear localization even in the presence of ELOVL6-IN-1 (Fig. 6b).

To further validate the effect of OEA on PPARα activation in TMEM135 depletion, we investigated whether OEA is a major metabolite to activate PPARα through lipid profiling under LO treatment. We observed that the OEA level was significantly increased at both 4 h and 12 h following LO administration (Supplementary Fig. 3b). By contrast, other known endogenous PPARα ligands, including PA and AA, did not show comparable changes under the same conditions. Notably, among the detected lipid species, OEA showed the most pronounced and sustained elevation at both time points, suggesting that it may have a prominent role in mediating PPARα activation in response to lipid challenge. Additionally, we performed PPRE-luciferase reporter assays in siCon and siTMEM135-transfected cells treated with ELOVL6-IN-1 and OEA. We confirmed that PPRE-luciferase activity was significantly increased in siTMEM135 cells compared with siCon (Fig. 6c). In particular, inhibition of ELOVL6 with ELOVL6-IN-1 markedly reduced this induction, indicating that endogenous lipid metabolism contributes to PPARα activation. Furthermore, treatment with OEA restored PPRE-luciferase activity in siTMEM135 cells even in the presence of ELOVL6 inhibitor (Fig. 6c). This observation suggests that OEA can directly activate PPARα in the ELOVL6 downstream pathway. Consistently, the expression of canonical PPARα target genes, such as *Acox1*, *Abcd3*, and *Cpt1α*, showed a similar pattern. Expression level was increased in siTMEM135 cells, suppressed by ELOVL6-IN-1, and restored by OEA treatment (Supplementary Fig. 3c). Taken together, these results demonstrate that PPARα transcriptional activity was increased in TMEM135 deficiency, and OEA is a key mediator contributing to PPARα activation.

Stearoyl-CoA desaturase 1 (SCD1), an enzyme involved in oleic acid synthesis and a precursor of OEA^34^, was increased in siTMEM135 cells and the intestine of TMEM135 KO mice (Fig. 6d,i). The increase in OEA levels induced by siTMEM135 was markedly suppressed by CAY10566, an SCD1 inhibitor (Fig. 6e). Similar to ELOVL6-IN-1 treatment, the increased FA uptake in siTMEM135 cells was reduced by LEI-401 (Fig. 6f). Conversely, the inhibition of FA uptake by ELOVL6-IN-1 was reversed by OEA treatment in siTMEM135 cells (Fig. 6g). The increased FA uptake by siTMEM135 was also attenuated by CAY10566 treatment (Fig. 6h). These results suggest that inhibition of OEA synthesis recapitulates the effects of ELOVL6 inhibition by reducing PPARα activation and FA uptake in TMEM135-deficient cells. In addition, we observed that SREBP1, one transcriptional factor that regulates ELOVL6, was activated in TMEM135-deficient intestine. Several target genes of SREBP1, such as *Acaca*, *Fasn*, *Acly*, and *Scd1*, were also upregulated in TMEM135 KO intestine (Fig. 6i and Supplementary Fig. 3d). Furthermore, we observed that *Srebf1* and the nuclear form, SREBP1N, were increased in siTMEM135 and TMEM135 KO mice (Fig. 6k and Supplementary Fig. 3d–f). Although SREBP1 was activated by TMEM135 depletion, mRNA and protein expression of ELOVL6 were increased. However, treatment with betulin, an SREBP1 inhibitor, decreased ELOVL6 mRNA and protein expression upregulated by siTMEM135 (Fig. 6j,k).

TMEM135 may be involved in peroxisomal lipid metabolism, where its depletion enhances β-oxidation through DHA depletion^7^. In this study, we focussed that TMEM135 would function as a transporter involved in the metabolism of fatty acids, particularly PUFAs such as DHA. To investigate this, structural prediction of TMEM135 was performed. According to the prediction model, TMEM135 adopts a membrane-embedded structure containing a putative pocket-like cavity, consistent with features commonly observed in transporter proteins (Fig. 6l,m). In addition, docking analysis suggested that DHA can interact with several residues within this pocket (Fig. 6n,o). Together, these observations support the possibility that TMEM135 may function as a transporter or carrier protein for DHA, thereby modulating intracellular lipid composition and downstream metabolic signaling. Furthermore, TMEM135 deficiency may promote DHA depletion, as suggested by Landowski et al.^7^. Reduced DHA levels have been reported to activate SREBP-1, thereby inducing the expression of lipogenic enzymes, including ELOVL6, which may contribute to the increased production of downstream lipid metabolites such as OEA^35,36^. Taken together, these data imply that PPARα is activated by OEA through ELOVL6, and SREBP1 regulates ELOVL6 in the intestine under TMEM135 deficiency.

### Inhibition of ELOVL6 attenuates FA uptake in the intestine of TMEM135 KO mice

**Fig. 7:**
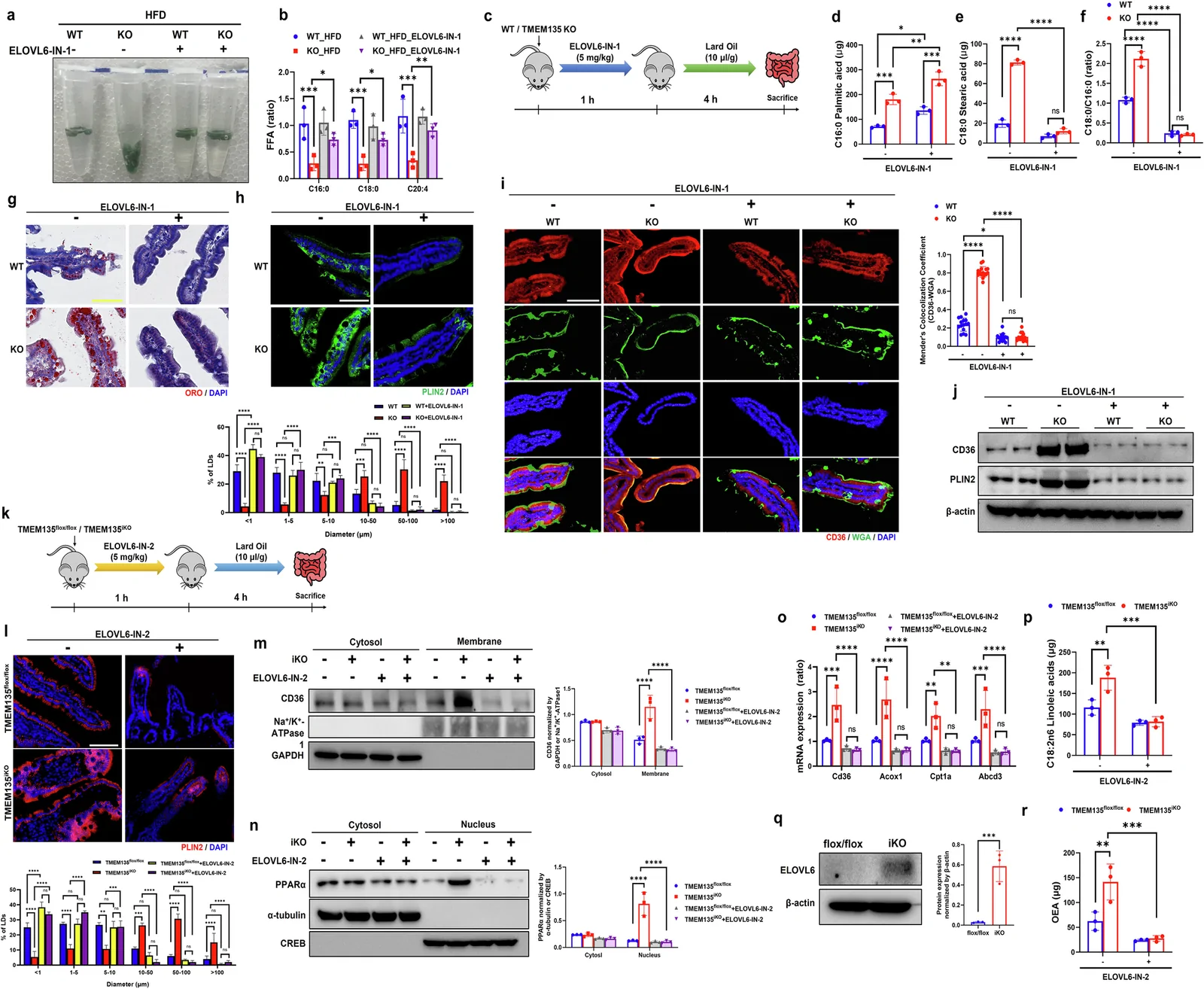
Effect of ELOVL6 on fatty acid uptake in the intestine of TMEM135-deficient mice after lard oil treatment. **a**,**b** WT and TMEM135 KO mice were fed either a normal chow diet or high-fat diet (HFD) for 12 weeks (*n* = 3 per group). ELOVL6-IN-1 (5 mg/kg) was administered to the mice once weekly for 3 weeks. **a** Feces floated on water. **b** Gas chromatography–mass spectrometry (GC–MS) analysis of palmitic acid (C16:0), stearic acid (C18:0), and arachidonic acid (C20:4) from feces of mice. **c**–**j** WT and TMEM135 KO mice were administered ELOVL6-IN-1 (5 mg/kg) for 1 h and then treated with lard oil (10 μl/g) for 4 h (*n* = 3 per group). **c** Experimental scheme. Analysis of palmitic acid (C16:0) (part **d**) and stearic acid (C18:0) (part **e**). **f** C18:0/C16:0 ratio. **g** Oil Red O (ORO) staining. Scale bar, 50 μm. **h** Immunofluorescence staining of PLIN2 and distribution of lipid droplet (LD) size. Scale bar, 50 μm. **i** Immunofluorescence staining of CD36 and WGA, and Menders’ colocalization coefficient. Scale bar, 50 μm. **j** Western blot and quantification for CD36, PLIN2, and β-actin. **k**–**r** TMEM135^flox/flox^ and TMEM135^iKO^ mice were administered ELOVL6-IN-2 (5 mg/kg) for 1 h and then treated with lard oil (10 μl/g) for 4 h (*n* = 3 per group). **k** Experimental scheme. **l** Immunofluorescence staining of PLIN2 and distribution of LD size. Scale bar, 50 μm. **m** Membrane fractionation and western blotting. CD36 was normalized by Na^+^/K^+^-ATPase1 (membrane) or GAPDH (cytosol) (*n* = 3). **n** Nucleus fractionation and western blotting. PPARα was normalized to α-tubulin or CREB (*n* = 3). **o** mRNA expression of PPARα target genes. **p** GC-MS analysis of linoleic acid (C18:2n6) (*n* = 3), **q** Western blot and quantification for ELOVL6 and β-actin. **r** GC–MS analysis of OEA (*n* = 3). All data represent mean ± SD, *P < 0.05, **P < 0.01, ***P < 0.001, ****P < 0.0001. DAPI 4′,6-diamidino-2-phenylindole, FFA free fatty acid, KO knockout, ns not significant, OEA oleoylethanolamide, PPARα peroxisome proliferator-activated receptor alpha, TMEM135 transmembrane protein 135, WT wild-type.

To verify the effect of ELOVL6 on lipid uptake in vivo, we used global TMEM135 KO mice and intestinal-specific TMEM135^iKO^ mice. First, we examined the effects of ELOVL6 on the feces of TMEM135 KO mice following a HFD. We administered ELOVL6-IN-1 to both WT and KO mice fed HFD, and we confirmed the effect of inhibitor using C18:0/C16:0 ratio (Supplementary Fig. 4m). As we previously observed, the feces of TMEM135 KO mice fed a HFD sank in water and did not float compared with the feces of WT mice. However, after administering ELOVL6-IN-1, the feces of TMEM135 KO mice did not sink in water (Fig. 7a). Additionally, we analyzed that the reduced concentrations of PA, SA, and AA in the feces of TMEM135 KO mice were restored after the administration of ELOVL6-IN-1 (Fig. 7b). These results imply that FA reduction in TMEM135-deficient feces was reversed by ELOVL6 inhibition, which represents less FA uptake even in TMEM135 deficiency. Next, WT and TMEM135 KO mice were treated with ELOVL6-IN-1 before LO administration (Fig. 7c). ELOVL6-IN-1 treatment increased PA concentration while it decreased SA in WT and TMEM135 KO mice (Fig. 7d–f). Additionally, ELOVL6-IN-1 treatment reduced C18:0/C16:0 ratio in both mice (Fig. 7f) and inhibited ORO intensity in TMEM135 KO mice (Fig. 7g). Similarly, the larger LDs detected in TMEM135 KO mice were notably reduced by ELOVL6-IN-1 treatment (Fig. 7h). The increased colocalization of CD36 with WGA, a plasma membrane marker, was significantly reduced by ELOVL6-IN-1 treatment in TMEM135 KO mice compared with WT (Fig. 7i). CD36 expression also decreased in TMEM135 KO mice by ELOVL6-IN-1, accompanied by reduced PLIN2 expression (Fig. 7j). Consistent with in vitro results, ELOVL6-IN-1 treatment reduced the increased nuclear translocation of PPARα in TMEM135 KO mice compared with WT (Supplementary Fig. 4a). Furthermore, ELOVL6-IN-1 treatment abolished the upregulated expression of PPARα target genes, including *Cd36*, *Acox1*, *Cpt1a*, and *Abcd3*, in TMEM135 KO mice (Supplementary Fig. 4b). An increased level of linoleic acid (C18:2n6), which was only absorbed from diet, was markedly decreased by ELOVL6-IN-1 treatment in TMEM135 KO mice (Supplementary Fig. 4c). Additionally, the OEA level, which was higher in TMEM135 KO mice compared with WT, was also significantly decreased by ELOVL6-IN-1 treatment (Supplementary Fig. 4d). These data imply that ELOVL6 activates PPARα, which further enhances lipid uptake under TMEM135 depletion.

Next, to minimize potential systemic effects of TMEM135 deficiency, we used intestinal-specific cKO mice (Supplementary Fig. 4e–g). We also used an additional ELOVL6 inhibitor, ELOVL6-IN-2, to validate the intestinal specific effects of TMEM135 deficiency. TMEM135^flox/flox^ and TMEM135^iKO^ mice were treated with ELOVL6-IN-2 before LO administration (Fig. 7k). ELOVL6-IN-2 treatment increased PA but decreased SA level in both mice (Supplementary Fig. 4h,i) and further reduced C18/C16:0 ratio (Supplementary Fig. 4j). Enlarged LDs observed in the intestine of TMEM135^iKO^ mice were markedly diminished by ELOVL6-IN-2 (Fig. 7l and Supplementary Fig. 4k). Expression of CD36 and PLIN2, which was elevated in TMEM135^iKO^ mice, was also suppressed by ELOVL6-IN-2 (Supplementary Fig. 4l). Membrane localization of CD36 was increased in TMEM135^iKO^ mice, whereas its translocation was prevented by ELOVL6-IN-2 (Fig. 7m). Similarly, the nuclear translocation of PPARα and the upregulation of target genes in TMEM135^iKO^ mice were abolished by ELOVL6-IN-2 (Fig. 7n,o). Linoleic acid level was increased in TMEM135^iKO^ mice, which was reduced by ELOVL6-IN-2 (Fig. 7p). ELOVL6 expression and its downstream metabolite OEA were also increased in TMEM135^iKO^ mice, whereas the OEA level was suppressed by inhibitor treatment (Fig. 7q,r). Collectively, these findings indicate that the effects of TMEM135 deletion are primarily driven by intestinal cellular mechanisms rather than systemic alterations.

### TMEM135 depletion improves FAO in peroxisomes and mitochondria

**Fig. 8:**
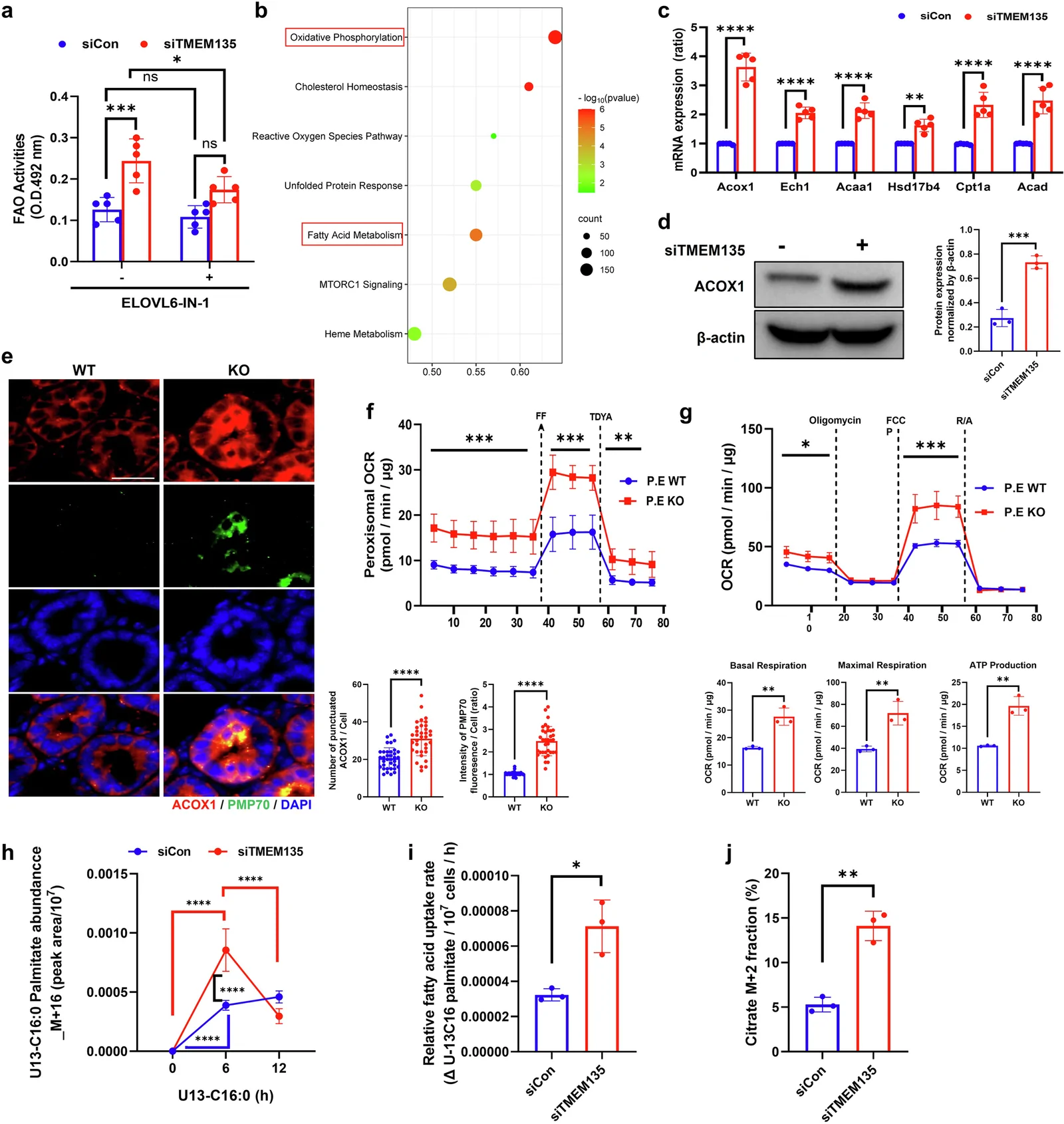
Effect of enhanced fatty acid oxidation on lipid content under TMEM135 depletion. **a** Fatty acid oxidation in Fhs74int cells treated with ELOVL6-IN-1 (10 μM) for 1 h and transfected with siCon or siTMEM135, *n* = 5 independent experiments. **b** Gene set enrichment analysis of RNA-seq from the intestine of WT and TMEM135 KO mice (*n* = 3). **c** mRNA expression in Fhs74int cells transfected with siCon or siTMEM135, *n* = 5 independent experiments. **d** Western blot and quantification for ACOX1 and β-actin in Fhs74int cells transfected with siCon or siTMEM135, *n* = 3 independent experiments. **e** Immunofluorescence staining and quantification for ACOX1 and PMP70. Scale bar, 30 μm. **f** Oxygen consumption rate (OCR) from isolated peroxisomes (peroxisomal OCR), *n* = 3 independent experiments. **g** OCR of the primary epithelial cells, basal respiration, maximal respiration, and ATP production, *n* = 3 independent experiments. **h**–**j** Fhs74Int cells were transfected with siCon or siTMEM135 for 6 h, followed by incubation with U-¹³C₁₆-palmitate, *n* = 3. Cells were harvested at 6 h and 12 h after U-¹³C₁₆-palmitate treatment. **h** Intracellular U-¹³C₁₆-palmitate abundance was quantified by liquid chromatography–mass spectrometry. **i** Fatty acid uptake rate of 6 h was calculated based on the time-dependent incorporation of U-¹³C₁₆-palmitate. **j** Fractional enrichment of citrate *m* + 2 in Fhs74Int cells transfected with siCon or siTMEM135 after 12 h treatment with U-¹³C₁₆-palmitate, *n* = 3. All data represent mean ± SD, *P < 0.05, **P < 0.01, ***P < 0.001, ****P < 0.0001. DAPI 4′,6-diamidino-2-phenylindole, FAO fatty acid oxidation, KO knockout, PMP peroxisomal membrane protein, TDYA trichosadiynoic acid, TMEM135 transmembrane protein 135, WT wild-type.

We speculated that the decreased intestinal lipid content in TMEM135 KO mice 12 h after LO treatment compared with WT, despite increased lipid uptake in the early phase of LO challenge, might be attributed to enhanced peroxisomal and mitochondrial function. FAO was increased in siTMEM135 cells and significantly decreased by ELOVL6-IN-1 treatment (Fig. 8a). GSEA of RNA-seq data revealed that genes upregulated in TMEM135 KO mice compared with WT mice were enriched in pathways related to oxidative phosphorylation (OXPHOS) and FA metabolism (Fig. 8b). mRNA and protein expression related to β-oxidation and OXPHOS were increased in siTMEM135 cells (Fig. 8c,d). Notably, the expression of ACOX1 and peroxisomal membrane protein 70 (PMP70), a peroxisomal protein which indicated peroxisomes, was markedly increased and colocalized in TMEM135 KO mice intestine (Fig. 8e). In primary epithelial cells of TMEM135 KO mice, peroxisomal FAO was enhanced under all conditions, including basal, FFA treatment, and treatment with the ACOX1 inhibitor, TDYA (Fig. 8f). Additionally, mitochondrial OXPHOS parameters were increased in primary epithelial cells from TMEM135 KO mice compared with WT mice (Fig. 8g).

Next, we evaluated fatty acid uptake and oxidation flux with U-¹³C₁₆-palmitate tracing. Fhs74Int cells transfected with siCon or siTMEM135 were treated with U-¹³C₁₆-palmitate. As a result, intracellular U-¹³C₁₆-palmitate level in siTMEM135 cells was significantly increased after 12 h of treatment compared with siCon cells (Fig. 8h). Similarly, the fatty acid uptake rate calculated based on intracellular U-¹³C₁₆-palmitate level over time was also significantly increased in siTMEM135 cells (Fig. 8i). These results suggest that TMEM135 deficiency increases fatty acid uptake. In addition, we quantified the *m* + 2 isotopolog fraction of citrate derived from U-¹³C₁₆-palmitate to evaluate FAO. During the β-oxidation process, labeled acetyl-CoA generated from U-¹³C₁₆-palmitate enters the tricarboxylic acid cycle and contributes to form *m* + 2 citrate^37^. We found that the citrate *m* + 2 fraction was significantly increased in siTMEM135-transfected cells compared with siCon (Fig. 8j). These results indicate that fatty acid-derived carbons are more actively oxidized and incorporated into the tricarboxylic acid cycle in TMEM135-deficient cells.

Collectively, these results suggest that enhanced lipid oxidation under TMEM135 depletion may contribute to preventing intestinal lipid accumulation through increased lipid metabolism.

## Discussion

TMEM135 has been implicated in lipid metabolism and utilization across multiple tissues^4^. Consistent with previous reports showing reduced hepatic lipid accumulation in TMEM135^FUN025/ob^ mice^7^ and lipid accumulation in brown adipose tissue of cKO mice owing to impaired mitochondrial fission^38^, our findings suggest that TMEM135 has a context-dependent role in lipid regulation. Following a 4 h LO challenge, TMEM135 deficiency enhanced CD36-mediated lipid absorption and transiently increased lipid content. However, 12 h of LO treatment or under HFD conditions, lipid accumulation was reduced, accompanied by elevated lipid oxidation in the intestine. These results indicate that loss of TMEM135 initially facilitates lipid uptake during early nutrient exposure but subsequently promotes lipid utilization through enhanced oxidative metabolism. Notably, the feces of TMEM135-deficient mice fed a HFD sank in water, which is opposite phenotype of inflammatory bowel disease (IBD)^39^. Increased LCFAs and polyunsaturated fatty acids were observed in the feces of patients with IBD^40^. Lipid absorption in the small intestine is a very important and highly adaptable process; however, if this process is overloaded by a HFD, it contributes significantly to the development of obesity and related metabolic diseases^41^. However, no significant lipid accumulation was observed in TMEM135 KO mice, despite being fed a HFD. Accordingly, we propose that TMEM135 may function as a key regulator of intestinal lipid absorption and metabolism.

A symptom of IBD, such as CD and ulcerative colitis, is malabsorption of nutrients in intestine^42^. The cause of IBD is still unknown, but many reports expect that it might be related with genetic backgrounds and excessive immune system against digestion^42^. Several studies have been reported to identify the causes of IBD using bulk RNA-seq and single-cell RNA-seq analysis. Among several differential gene expressions, TMEM135 ranks highly in several IBD models. TMEM135 is considered as a risk factor gene through genome-wide association studies in patients with IBD^43,44^. In addition, TMEM135 was also introduced as a methylated gene in IBD^45^. These reports have clarified that TMEM135 may be a risk factor on patients with IBD. Although an increase in expression of TMEM135 has been observed in various intestinal tissues of patients with various types of IBDs, it cannot be concluded that TMEM135 is a major cause of IBD. However, the possibility that TMEM135 is associated with IBD symptoms is worth considering.

In addition, we investigated whether TMEM135 expression is associated with CD using publicly available RNA-seq data from patients with CD^27^ as intestinal epithelial dysfunction in CD is closely linked to changes in fatty acid metabolism. As a results, heatmap analysis revealed that TMEM135 expression in patients with CD was upregulated when compared with non-inflammatory bowel disease (non-IBD) control groups (Supplementary Fig. 5a). Volcano plot analysis also confirmed a significant increase in TMEM135 expression in patients with CD (Supplementary Fig. 5b). Among ranking of the most highly upregulated genes, TMEM135 was included among the top 40 genes in patients with CD (Supplementary Fig. 5c). In GSEA, adipogenesis-related pathways were markedly enriched in patients with CD, which indirectly supports a significant change in lipid metabolic activity (Supplementary Fig. 5d). Collectively, these findings demonstrate that TMEM135 is elevated in CD and suggest its potential clinical relevance in pathological intestinal lipid absorption.

The main lipid transporter in the apical membrane of intestinal epithelial cells is CD36 (refs. ^46,47^). In PPARα-deficient mice, FA uptake was reduced, as was CD36 expression^48^. In this study, GW6471 treatment reduced LCFA uptake accompanied by a reversal CD36 expression and membrane localization. Another major FA transporter is the FATP family proteins^49^. Among the various isoforms, FATP4 is the predominant form in the intestine^49^. However, FATP4 deficiency did not significantly reduce FA uptake in TMEM135-deficient cells. Several explanations for this observation are possible. First, because FATP4 is not a direct target gene of PPARα, PPARα-induced FA uptake may not be affected by FATP4 deficiency in TMEM135 depletion. Second, FATPs may have a more important role in the transport of VLCFAs than LCFAs. Furthermore, FATP4 may have only a minor role among the six FATP isoforms in the intestine. Therefore, we consider PPARα to be a crucial transcriptional factor that increases lipid absorption through upregulation of CD36 in TMEM135-deficient intestines.

PPARα is a transcriptional factor, which is ligand-dependently activated in the nucleus^50^. Furthermore, the pool of PPARα resides in the cytosol and can translocate to the nucleus upon ligand binding^51–54^. Our data showed that cytoplasmic PPARα localizes to the nucleus and functions as a transcription factor. SA elongated by ELOVL6 is converted to OEA, which acts as a ligand for PPARα activation^55^. OEA activates PPARα in the intestine, liver, adipocytes, and muscles to upregulate CD36 (refs. ^56–59^). OEA administration improves metabolic flexibility and prevents obesity^60^. Therefore, we propose that cytosol-synthesized OEA driven by ELOVL6 in TMEM135-deficient cells binds to this cytosolic PPARα pool, promoting its nuclear translocation and subsequent transcriptional activation.

ELOVL6 expression is regulated by several transcription factors, and SREBP1 directly targets the ELOVL6 promoter^61^. SREBP1 target genes were upregulated, and *Srebf1* expression and proteolytic cleavage of SREBP1 were increased upon TMEM135 depletion (Supplementary Fig. 3d,e). Furthermore, *Rhbdl4*, which cleaves SREBP1, was also upregulated in siTMEM135, whereas *Chrebp*, *Srebf2*, and their target genes did not change in response to TMEM135 deficiency (Supplementary Fig. 3d,f) SREBP1 activation is crucial for inducing ELOVL6 expression. Decreased DHA levels contribute to activate SREBP1 (ref. ^35^) and reduced DHA levels have been reported in TMEM135 loss-of-function mutant mice^7^. We observed a significant reduction in DHA (C22:6) level at 4 h and 12 h of LO challenge in TMEM135-deficient intestine (Supplementary Fig. 3b). These findings suggest that ELOVL6 is upregulated by SREBP1 in TMEM135-deficient intestine.

Peroxisome abundance may explain the contradictory phenotypes observed in ELOVL6-deficient mice. ELOVL6 deficiency protects against obesity and hepatic lipid accumulation^62,63^ and enhanced ELOVL6 activity promotes LD formation^13^. TMEM135 depletion increased ELOVL6 activity and lipid uptake, but elevated peroxisome abundance offsets these effects by enhancing β-oxidation, thereby preventing lipid accumulation. Unlike ELOVL5, which was upregulated in TMEM135^FUN025/FUN025^ liver^7^, *Elovl5* expression remained unchanged in TMEM135 KO intestine. As ELOVL6 predominantly metabolizes monounsaturated FAs^64^ and our diets were enriched in oleic and PAs rather than linoleic acid, tissue-dependent and diet-dependent activation of specific ELOVL isoforms likely accounts for these differences.

We investigated whether TMEM135 regulates lipid content via chylomicron secretion using a Transwell system in Caco-2 cells transfected with siCon or siTMEM135 (Supplementary Fig. 6a,b). Upon oleic acid treatment, chylomicron-associated ApoB secretion was detected in siCon and siTMEM135 cells (Supplementary Fig. 6c). ApoB concentration in the apical medium remained unchanged, whereas basolateral ApoB increased similarly in both cells, indicating no significant difference in chylomicron secretion (Supplementary Fig. 6c). Although sulfo-*N*-succinimidyl oleate, GW6471, and TDYA did not affect chylomicron secretion between siCon and siTMEM135 cells, sulfo-*N-*succinimidyl oleate or GW6471 treatment reduced lipid uptake in TMEM135-depleted cells (Supplementary Fig. 6d–f). Furthermore, expression of *Mgat* and *Dgat*, encoding key enzymes for monoacylglycerol and diacylglycerol conversion during chylomicron and LD formation^65^, was significantly increased upon TMEM135 depletion (Supplementary Fig. 6g). This upregulation may result from SREBP1 activation triggered by DHA depletion in TMEM135 deficiency. By contrast, *Mttp* expression, crucial for chylomicron assembly, was unchanged (Supplementary Fig. 6g). In vivo, serum TG levels increased 4 h after LO challenge in both WT and TMEM135 KO mice, without significant difference between the two groups. TG levels decreased 12 h after LO compared with 4 h, again without genotype differences (Supplementary Fig. 6h). Similarly, serum ApoB48, a chylomicron marker, did not differ significantly between WT and TMEM135 KO mice at either 4 h or 12 h, following a pattern similar to TG levels (Supplementary Fig. 6i). Collectively, these data indicate that chylomicron secretion did not significantly contribute to the lipid reduction observed 12 h after LO challenge in TMEM135 KO mice intestine.

β-oxidation may elevate lipid uptake in TMEM135-deficient intestines. Lipid oxidation is important for maintaining intestinal function, as the intestines consume 25% of oxygen in the process of absorbing nutrients from food and delivering them to other tissues^66^. Enhanced lipid absorption promotes chylomicron secretion by intestinal epithelial cells^67^. An imbalance between lipid absorption and oxidation may lead to lipid-related stress^17^. Treatment with etomoxir, a mitochondrial FAO inhibitor, induces intestinal PA absorption^48^. In TMEM135 KO mice, FAO was significantly increased, and lipid content was consistently reduced without changes in chylomicron secretion. This state enhances lipid absorption mechanisms to maintain intestinal lipid content at a constant level.

Several factors may underlie the simultaneous induction of peroxisomal and mitochondrial FAO in TMEM135-deficient mice. Peroxisomal FAO, primarily LCFAs and VLCFAs, is subsequently transferred to the mitochondria^68^. PPARα activation may also coordinate this process, as TMEM135 deficiency upregulates *Acox1* and *Cpt1a*, key PPARα target genes involved in FAO. We further identified ELOVL6 as a critical mediator linking peroxisomal and mitochondrial oxidation. ELOVL6 inhibition abolished the increased FAO and OXPHOS activity caused by TMEM135 deficiency (Supplementary Fig. 7a,b). Consistently, TMEM135-depleted cells had elevated C22:0 levels, which were reduced by ELOVL6 inhibitor (Supplementary Fig. 7a). As ELOVL6 elongates C16:0 to C18:0, generating precursors for OEA synthesis and VLCFA oxidation, enhanced ELOVL6 activity may activate PPARα, thereby promoting both peroxisomal and mitochondrial β-oxidation. Together, these findings suggest that TMEM135 deficiency elicits an adaptive metabolic response, in which ELOVL6-dependent lipid elongation and PPARα activation cooperatively enhance FAO to maintain lipid homeostasis.

Our previous study showed that CD36 expression was suppressed by SIRT1 in the liver of TMEM135 KO mice^69^, whereas this study showed that expression was increased in the intestine, indicating tissue-specific regulation. Four factors may explain this discrepancy. First, ELOVL6 expression was markedly upregulated in the intestine but not in the liver. ELOVL2 was induced in the liver (Supplementary Fig. 7c), suggesting a minimal role for ELOVL6 in the liver. Second, SIRT1 expression, which downregulates CD36, remained unchanged after 4 h of LO treatment; however, it was strongly induced after 12 h (Supplementary Fig. 7d). Correspondingly, CD36 and FA uptake increased at 4 h and decreased at 12 h (Fig. 3a), coinciding with increased SIRT1 activity and NAD⁺/NADH ratio in TMEM135 KO intestine (Supplementary Fig. 7e,f). These findings suggest that SIRT1 suppresses CD36 expression during the late phase of LO treatment. Third, CD36 protein is highly abundant and sensitively regulated in the intestine compared with other tissues (Supplementary Fig. 7g). Finally, consistent results were observed in both TMEM135 KO and TMEM135^iKO^ mice, confirming tissue-specific effects rather than systemic influences (Fig. 7). These results suggest that CD36-mediated lipid uptake is differentially regulated by TMEM135 between the liver and intestine in a tissue-dependent manner.

We directly evaluated intestinal permeability using an FITC–dextran assay in WT and TMEM135 KO mice, in the presence or absence of LO. FITC–dextran was administered orally, and plasma fluorescence intensity was measured. We found no significant differences in FITC–dextran level between WT and KO mice under either condition (Supplementary Fig. 7h), indicating that intestinal permeability was not altered by TMEM135 deficiency. To further evaluate epithelial integrity, we examined tight junction organization and protein expression. Immunofluorescence staining of ZO-1 revealed a characteristic honeycomb-like pattern along the epithelial layers, with no detectable discontinuities in both WT or KO mice (Supplementary Fig. 7i). This structural organization was preserved regardless of LO treatment. In addition, protein expression levels of key tight junction components, including ZO-1 and occludin, were comparable between WT and KO mice under all conditions tested (Supplementary Fig. 7j). Taken together, these results indicate that TMEM135 deficiency does not impair any of intestinal barrier function and tight junction integrity under LO challenge. In particular, these findings suggest that the enhanced fatty acid uptake and oxidation observed in TMEM135-deficient intestine do not compromise epithelial integrity.

Owing to the high hydrophobicity of TMEM135, elucidating its structure and function remains challenging. TMEM135 may function as a DHA transporter or bind to AKAP for mitochondria fission^7,38^. We speculated that these functions may also be associated with the phenotypes observed in this study. However, enzymatic studies are required to clarify the precise role of TMEM135 in intestinal lipid absorption and FAO.

In summary, we provide the first evidence that TMEM135 regulates lipid uptake and metabolism in the intestine. TMEM135 depletion promotes ELOVL6 expression, activates PPARα, and increases CD36 expression on the plasma membrane, thereby enhancing lipid uptake. Increased expression and activity of ELOVL6 induce OEA, further activating PPARα and influencing peroxisome abundance in TMEM135-deficient intestines. Absorbed lipids are then oxidized in the peroxisomes and mitochondria as metabolic substrates. Consequently, increased metabolic activity maintains lipid homeostasis by preventing lipid accumulation. Together, our findings highlight the pivotal role of TMEM135 in coordinating lipid absorption and maintaining lipid balance in the intestine.

## Supplementary information

## Data Availability

RNA-seq data have been deposited in Gene Expression Omnibus (GEO) (Accession No. GSE291662).
