# Supplementary material for: TMEM135 deficiency prevents intestinal lipid accumulation by coordinating lipid uptake and oxidation through ELOVL6-mediated PPARα activation
Source: Exp Mol Med. 2026 Aug 5;58(8):2654–69. doi: 10.1038/s12276-026-01795-z (PMC13538681; doi:10.1038/s12276-026-01795-z)
Supplement: Supplementary file 1 [file 12276_2026_1795_MOESM1_ESM.pdf]

Supplementary Materials for

TMEM135 Prevents Intestinal Lipid Accumulation by Coordinating Lipid Uptake and Oxidation through ELOVL6-Mediated PPARα Activation

Hyunsoo Kim[1], Channy Park[1], Xiaofan Wei[1], Arun Chhetri[1], Laxman Manandhar[1], Jiwon Seo[2], Yoonkyung Park[3], Sang-Wook Lee[4], and Raekil Park[1*]

1 Department of Biomedical Science and Engineering, Gwangju Institute of Science and Technology, Gwangju, Republic of Korea

2 Department of Chemistry, Gwangju Institute of Science and Technology, Gwangju, Republic of Korea

3 Department of Biomedical Science, Chosun University, Gwangju, Republic of Korea

4 Department of Radiation Oncology, Asan Medical Center, Seoul, Republic of Korea

* Correspondence to:

Raekil Park, M.D, Ph.D.

rkpark@gist.ac.kr

Department of Biomedical Science & Engineering,

Gwangju Institute of Science and Technology, Gwangju 61005, Republic of Korea

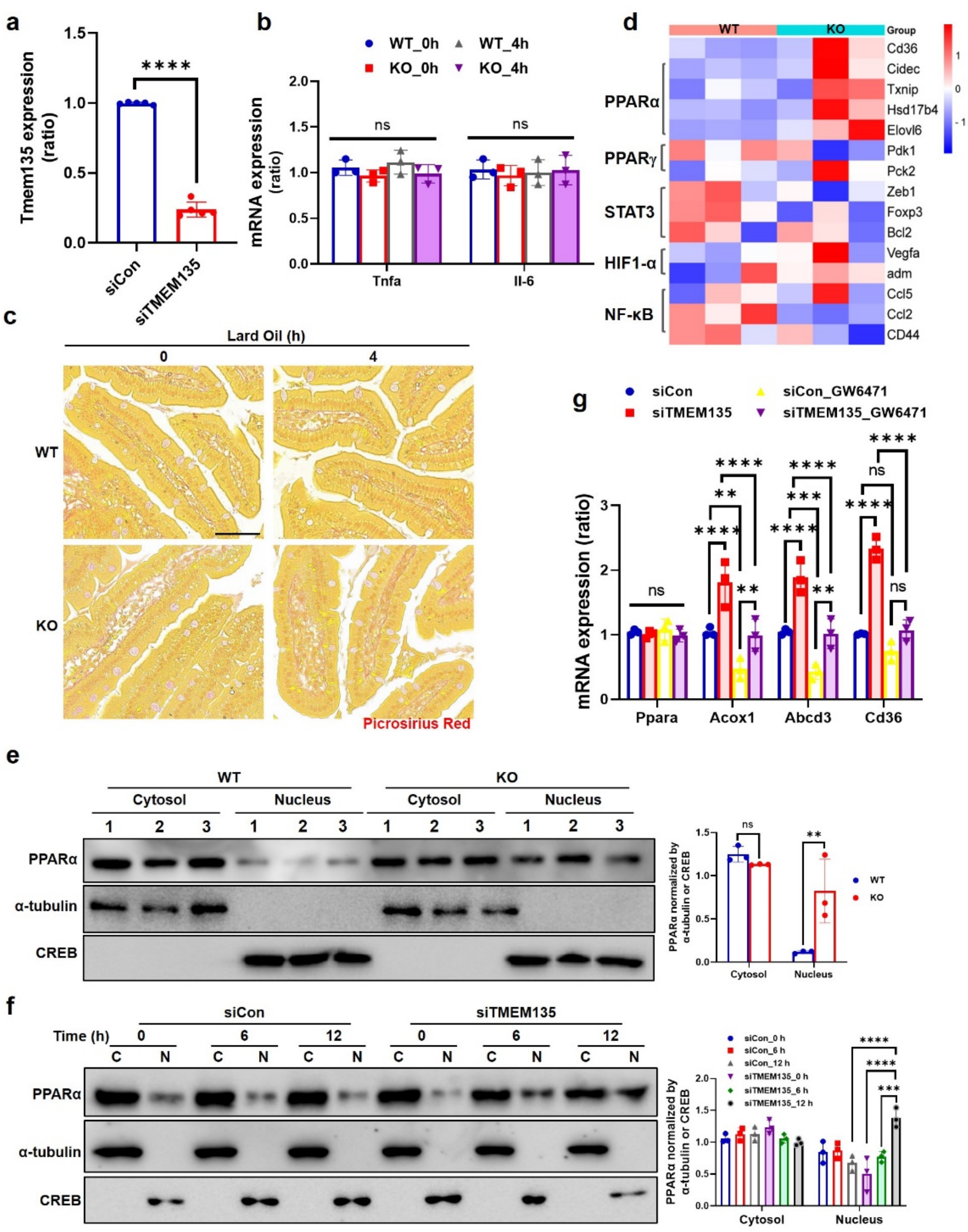

Supplementary Fig. 1. PPARα is activated under TMEM135 depletion conditions.

a TMEM135 mRNA expression in Fhs74int cells transfected with siCon or siTMEM135. *n* = 5, independent experiments. **b-c** WT and TMEM135 KO mice were treated with lard oil for 0 h and 4 h (*n* =3 per group)**. b** mRNA expression levels of inflammatory markers. **c** Picrosirius Red staining of intestine. **d** Heatmap of Bulk RNA Seq analysis showed expression of several transcriptional target genes in the intestine of WT and TMEM135 KO mice. *n* = 3. **e** Nuclear localization of PPARα was shown with α-tubulin (cytosolic marker) and CREB (nuclear marker) in the intestine of WT and KO.

Protein expression was normalized to α-tubulin or CREB. $n = 3$, independent experiments. **f** Nuclear translocation of PPARα was observed during transfection of Fhs74int cells with either siCon or siTMEM135 in a time-dependent manner. Protein expression was normalized to α-tubulin or CREB. $n = 3$, independent experiments. **g** PPARα and its target genes mRNA expression were analyzed using qPCR in the indicated conditions. $n = 3$. All data represent the mean ± SD, **$p<0.01$, ***$p<0.001$, ****$p<0.0001$.

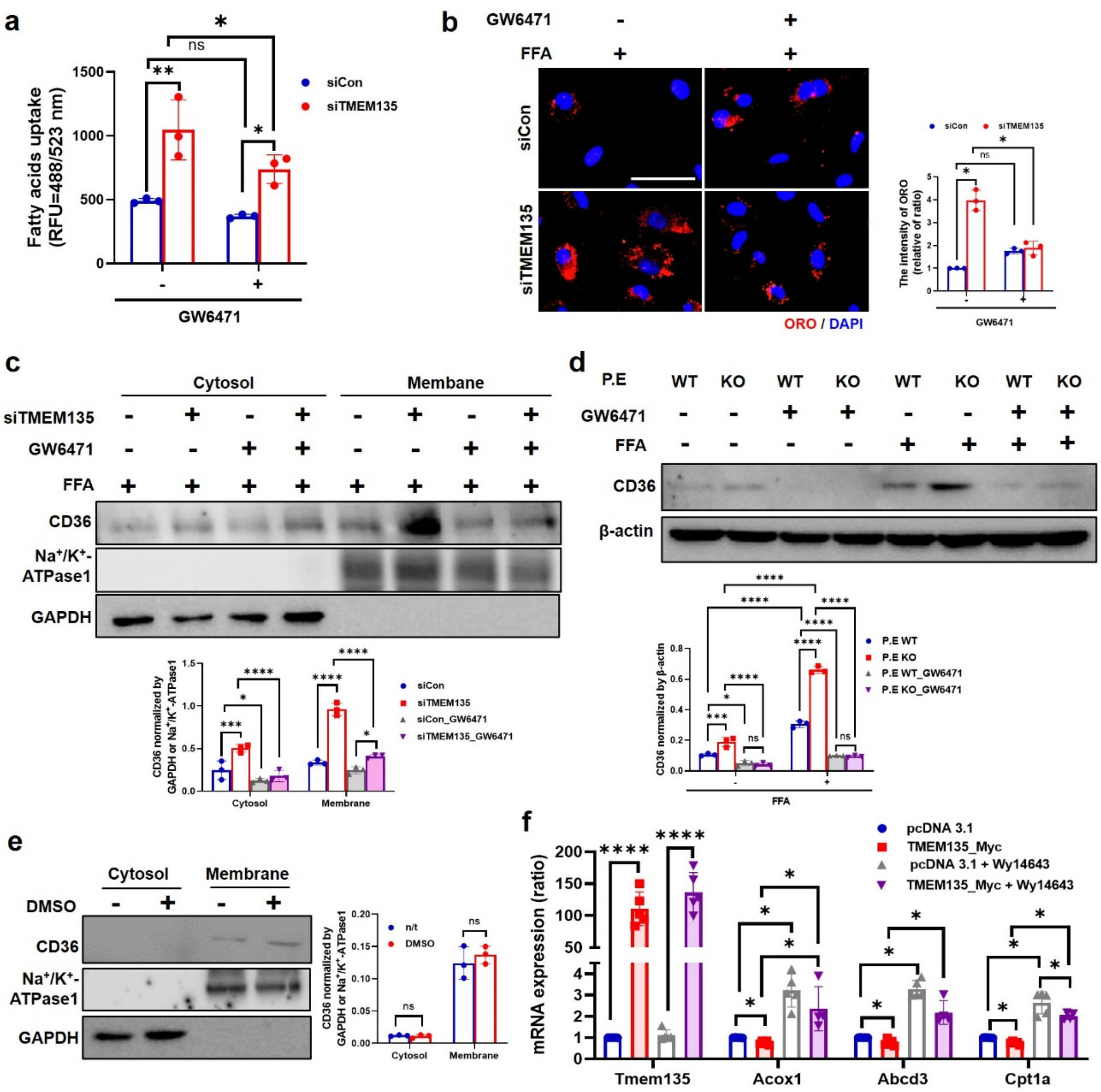

Supplementary Fig. 2. PPARα antagonist modulates lipid uptake in TMEM135 depletion and overexpression conditions.

a-c Fhs74int cells were transfected with siCon and siTMEM135 for 12 h after treatment with GW6471 (5 μM) for 1 h and **b-c** free fatty acid (FFA) (200 μM). **a** Fatty acid uptake assay. *n* = 3, independent experiments. **b** Oil Red O staining and quantification. Scale bar, 10 μm. *n* = 3, independent experiments. **c** Membrane fractionation, western blot and quantification for CD36 and Na^+/K^+-ATPase1 (membrane) or GAPDH (cytosol), *n* = 3, independent experiments. **d** Primary epithelial cells isolated from the intestine of WT and TMEM135 KO mice were treated with GW6471 (5 μM) for 1 h (*n* = 3). Western blot and quantification for CD36 and β-actin. **e** Membrane fractionation, western blot and quantification for CD36 and Na^+/K^+-ATPase1 (membrane) or GAPDH (cytosol) after treatment of

DMSO, $n = 3$, independent experiments. **f** Fhs74int cells were overexpressed with TMEM135-myc and pre-treated with WY-14643 (25 μM) for 1 h. mRNA expression of TMEM135 and PPARα target genes was analyzed by qPCR. $n = 5$, independent experiments. All data represent the mean ± SD, *p<0.05, **p<0.01, ***p<0.001, ***p<0.0001. All data represent the mean ± SD, *p<0.05, **p<0.01, ***p<0.001, ***p<0.0001.

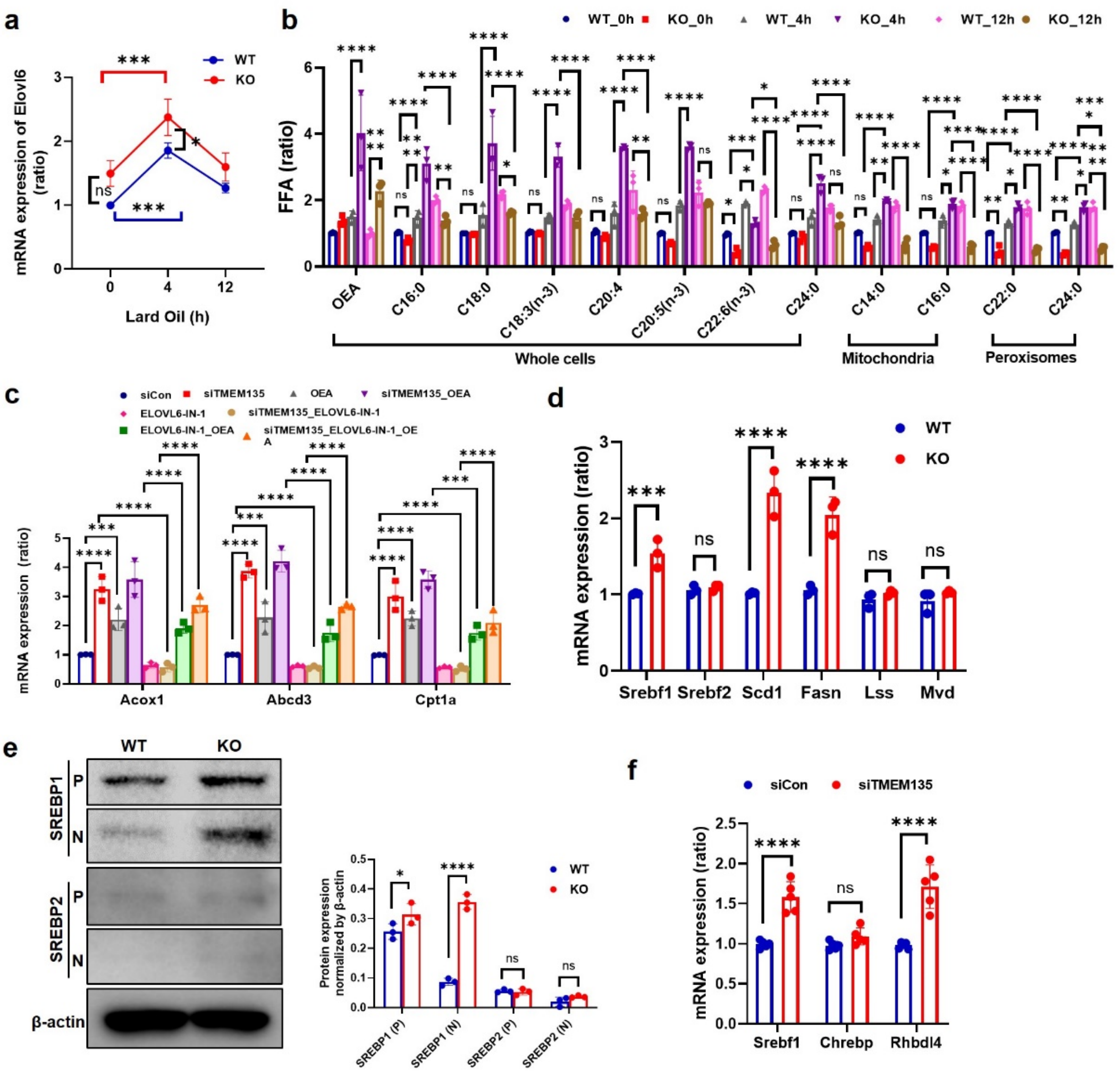

Supplementary Fig. 3. ELOVL6 is regulated by SREBP1 activation in the intestine of TMEM135 KO mice.

a Mice were orally administrated with lard oil (10 µl/g body weight, *n* = 3 per group). *Elovl6* mRNA expression of WT and TMEM135 KO duodenum, *n* =3. **b** Lipidomic analysis of whole cells, mitochondria, and peroxisomes from lard oil administrated mice (*n* = 3). **c** Fhs74int cells were treated with ELOVL6-IN-1 (10 µM) for 1 h and transfected with siCon or siTMEM135 for 12 h. Transfected cells were treated with OEA (10 µM). mRNA expression was measured, *n* = 3. **d** mRNA expression of the WT and TMEM135 KO intestine (*n* =3). **e** Western blot and quantification for SREBP1, SREBP2, and β-actin. P and N represented before and after proteolytic cleavage of SREBP1 respectively (*n* = 3). **f** mRNA expression of siCon and siTMEM135 transfected Fhs74int cells. *n* = 5, independent experiments. All data represent the mean ± SD, *p<0.05, ***p*<0.01, ****p*<0.001, ****p*<0.0001.

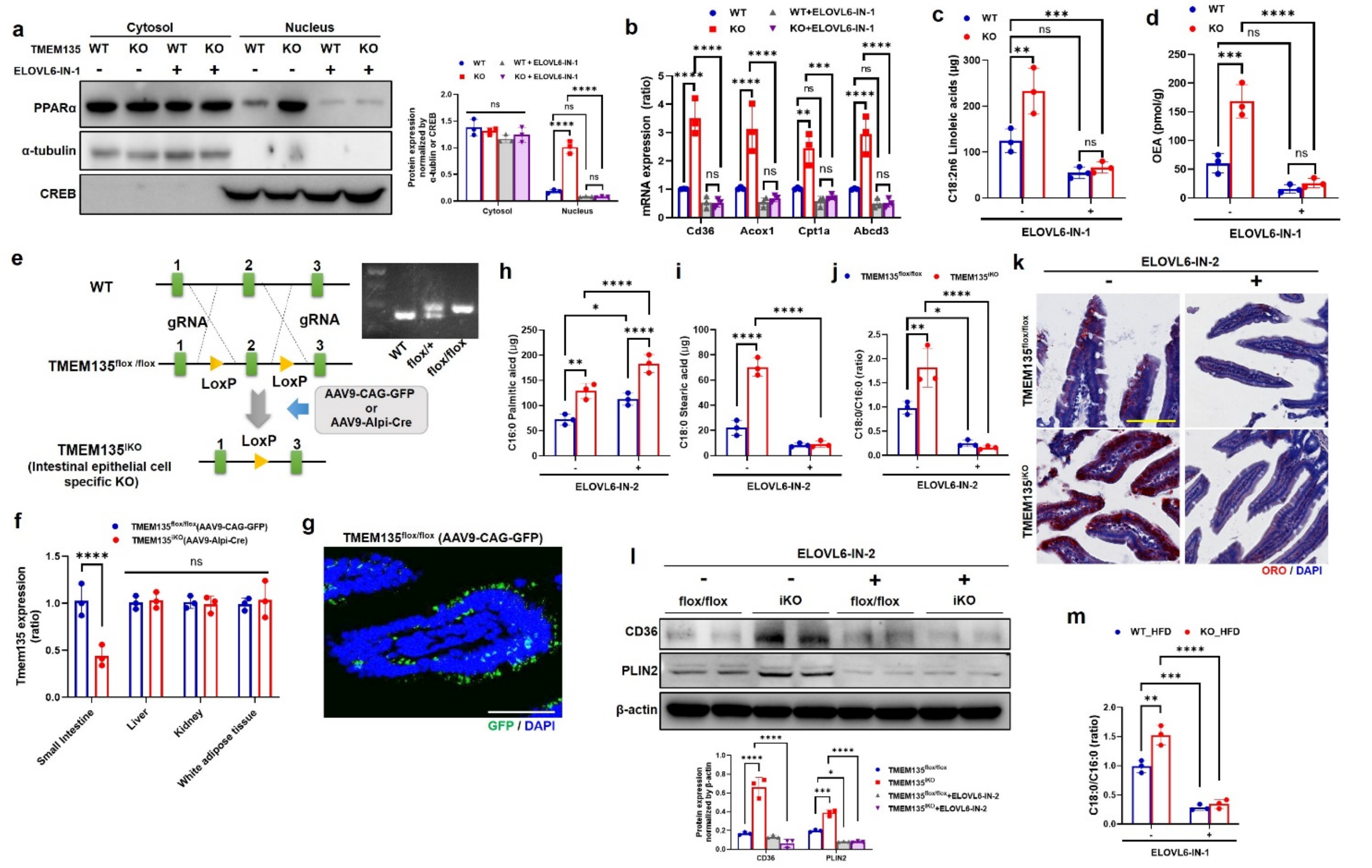

Supplementary Fig. 4. ELOVL6 inhibition suppresses FA uptake in TMEM135 deficiency mice.

a Nucleus fractionation, Western blot and quantification for PPARα and α-tubulin (cytosol) or CREB (nuclear fraction). **b** mRNA expression of PPARα target genes. GC-MS analysis of **c** linoleic acid (C18:2n6) (*n* = 3), and **d** OEA (*n* = 3). **e** Scheme for generating intestinal epithelial cell-specific knockout ($TMEM135^{iKO}$) mice by infecting flox/flox mice with AAV9-CAG-GFP or AAV9-Alpi-Cre. **f** *Tmem135* mRNA expression of several tissue after infection with AAV virus in $TMEM135^{flox/flox}$ or $TMEM135^{iKO}$ mice (*n* = 3). **g** AAV infection was visualized by GFP signals in the intestine. Scale bar, 50 μm. GC-MS analysis of **h** palmitic acid (C16:0) and **i** stearic acid (C18:0). **j** C18:0/C16:0 ratio. **k** Oil Red O staining. **l** Western blot and quantification for CD36, PLIN2 and β-actin. Scale bar, 50 μm. **m** WT and TMEM135 KO mice were fed either a normal chow diet (NCD) or high-fat diet (HFD) for 12 weeks (*n* = 3 per group). ELOVL6-IN-1 (5 mg/kg) was administered to the mice once weekly for 3 weeks. C18:0/C16:0 ratio of intestine were measured based on GC-MS analysis (*n* =3). All data represent the mean ± SD, *p<0.05, ***p*<0.01, ****p*<0.001, ****p*<0.0001.

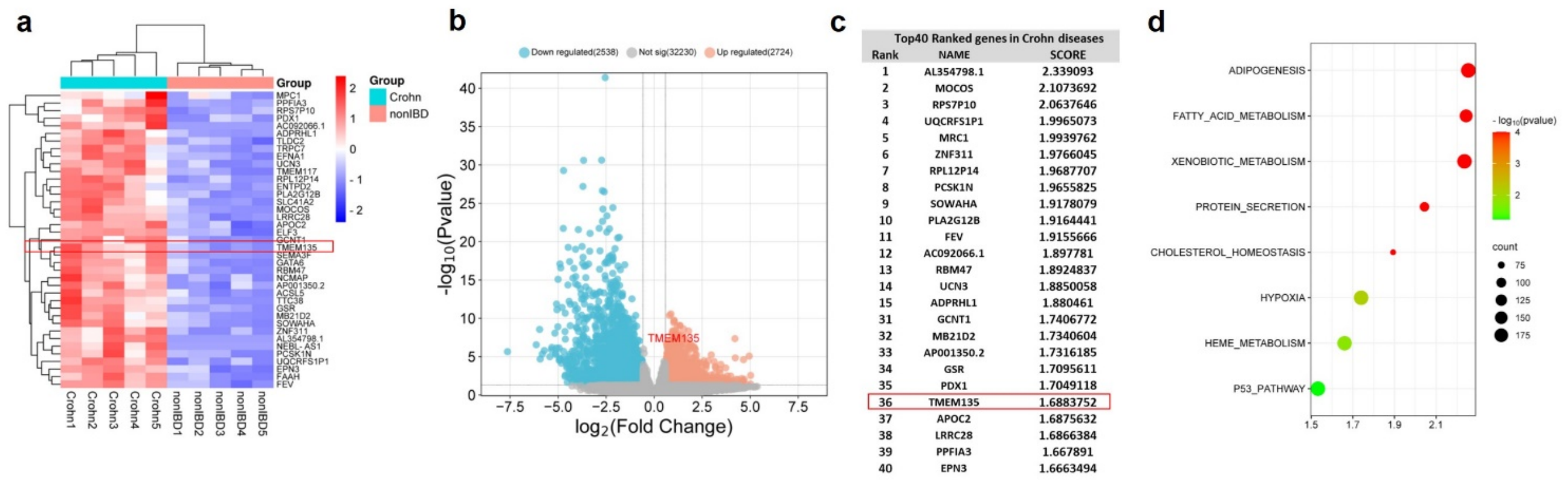

Supplementary Fig. 5. The expression of TMEM135 is increased in Crohn's disease model

a-d RNA seq samples from Crohn's diseases patients (*n* = 5) or nonIBD (*n* = 5) were analyzed. **a** Heatmap, **b** Volcano plot, **c** Ranked gene score, **d** Gene set enrichment analysis and bubble plot.

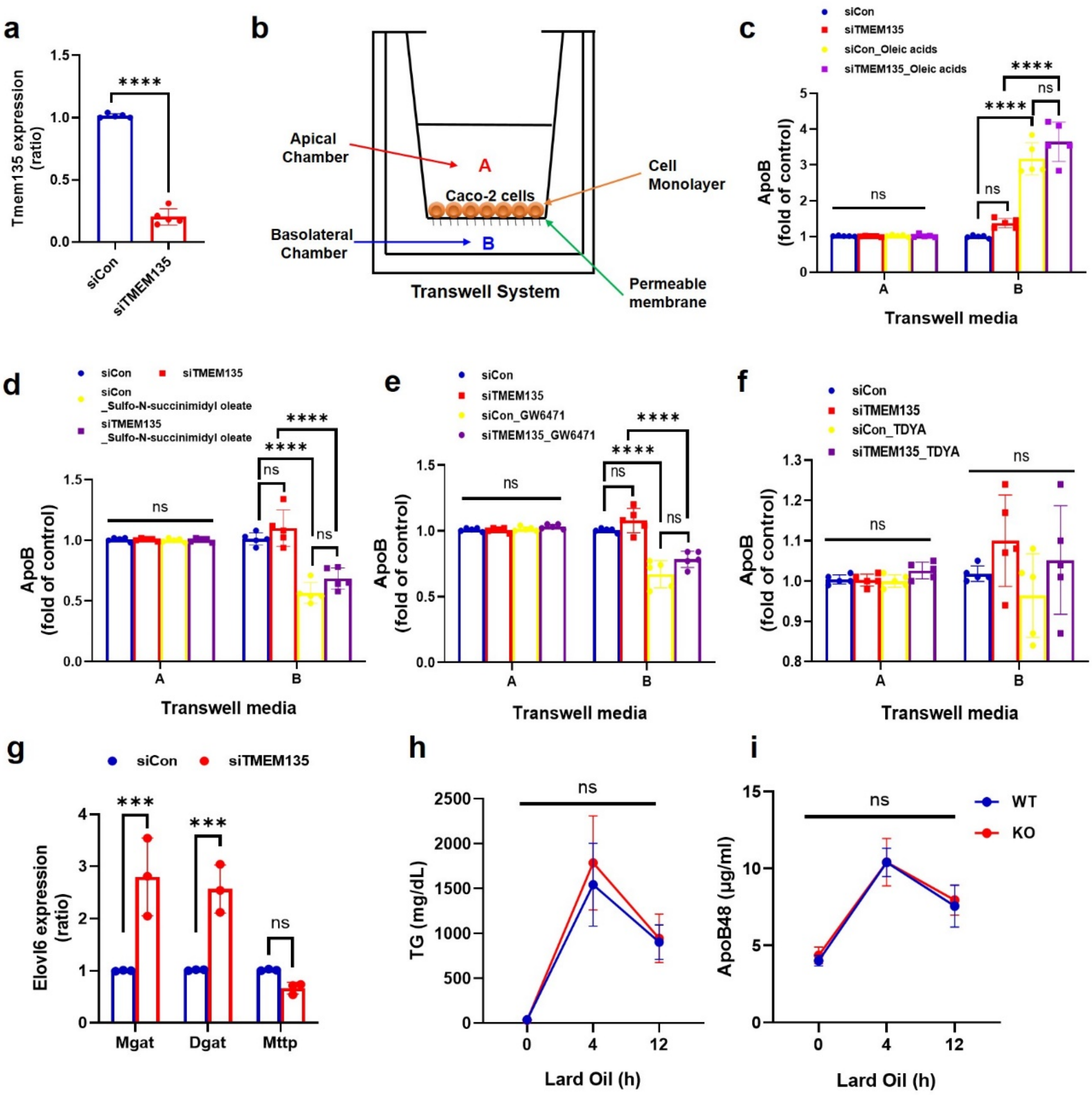

Supplementary Fig. 6. Chylomicron secretion is not affected in TMEM135 deficiency.

a mRNA expression of TMEM135 in Caco-2 cells transfected with siCon or siTMEM135 (n = 5). **b** Scheme of a Transwell system. **c-f** ApoB concentration from media in apical chamber A and basolateral chamber B was measured by ELISA in the indicated conditions. All data represent the mean ± S.D (n = 5, independent experiments), ****p<0.0001. **g** mRNA expression of siCon and siTMEM135 transfected Fhs74int cells. n = 3, independent experiments. **h-i** Mice were orally administrated with lard oil (10 μl/g body weight, n = 3, per group). **h** Triglyceride (TG) and **i** ApoB48 level in serum (n = 3). All data represent the mean ± SD, *p<0.05, **p<0.01, ***p<0.001, ***p<0.0001.

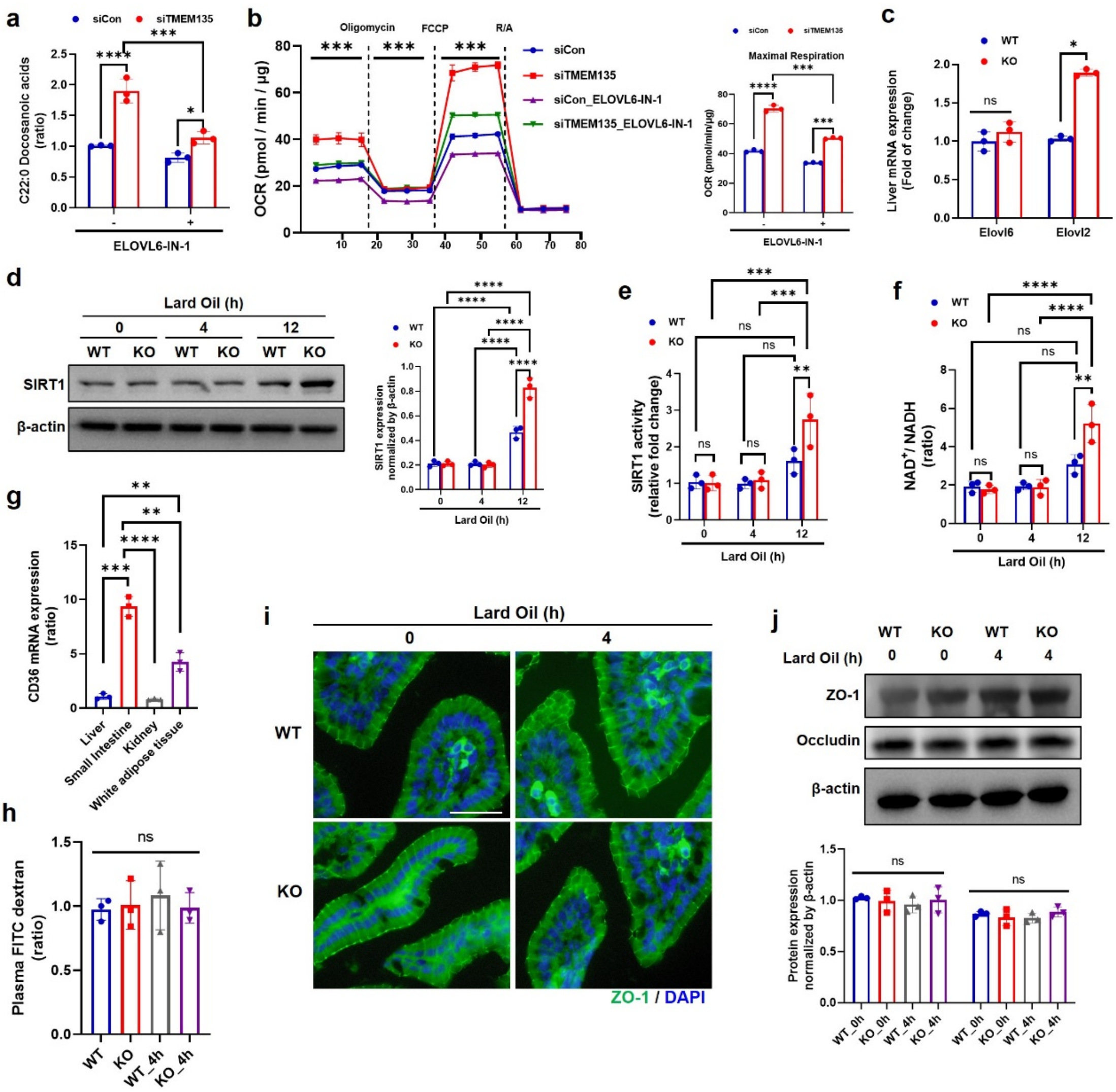

Supplementary Fig. 7. ELOVL6 is a crucial enzyme to induces FAO in TMEM135 deficiency.

a-b Fhs74int cells were treated with ELOVL6-IN-1 (10 µM) for 1 h and then, transfected with siCon or siTMEM135 for 12 h. **a** Docosanoic acids level was measured by GC-MS analysis ($n = 3$). **b** OCR and maximal respiration ($n = 3$). **c** mRNA expression of WT and TMEM135 KO mice liver ($n = 3$). **d-f** Lard oil administrated WT and TMEM135 KO mice ($n = 3$). **d** Western blot and quantification for SIRT1 and β-actin. **e** SIRT1 activity. **f** NAD^+/NADH ratio. **g** CD36 mRNA expression of several tissues were determined by qPCR ($n = 3$). **h-j** WT and TMEM135 KO mice were treated with lard oil for 0 h and 4 h ($n = 3$ per group). **h** Intensity of plasma FITC dextran was measured. **i** Intestines were stained with ZO-1. **j** Protein expression of ZO-1 and Occludin. All data represent the mean ± SD, *$p<0.05$, **$p<0.01$, ***$p<0.001$, ***$p<0.0001$.

Supplementary Tables

Supplementary Table 1. siRNA sequences

Targets	Sequence
Tmem135	5’- GCUCAAAUUAUGCAGUAGA -3’
	5’- AGUUCAGGACAGCUGUGAU -3’
Fatp4 (Slc27a4)	5’-CUCCAUCCAAAACUGCCAA-3’
	5’-UUGGCAGUUUUGGAUGGAG-3’

Supplementary Table 2. Antibodies

Proteins	Origin	Antibody details
β-actin	Mouse	Santa Cruz Biotechnology (sc-47778)
PLIN2	Mouse	Sigma-Aldrich (SAB4200452)
PLIN2	Rabbit	Proteintech (15294-1-AP)
I-FABP	Goat	Abcam (ab60272)
CD36	Rabbit	Abcam (ab133625)
FATP4	Rabbit	Abcam (ab200353)
SCD1	Rabbit	Cell Signalling (2794)
NA/K^+-ATPase1	Rabbit	Invitrogen (ST0533)
GAPDH	Rabbit	Cell Signalling (5174)
CREB	Rabbit	Cell Signalling (4820S)
α-tubulin	Mouse	Santa Cruz Biotechnology (sc-58666)
ELOVL6	Rabbit	Abcam (ab69857)
ELOVL5	Rabbit	Abcam (ab205535)
PPARα	Mouse	Santa Cruz Biotechnology (sc-398394)
WGA	Wheat	Invitrogen (W11261)
ACOX1	Rabbit	Proteintech (10957-1-AP)
PMP70	Mouse	Sigma-Aldrich (SAB4200181)
Myc	Rabbit	Cell Signaling (2278T)
Alexa Flour 488 goat anti-rabbit IgG (H+L)	Goat	Invitrogen (A11034)
Alexa Flour 568 goat anti-mouse IgG (H+L)	Goat	Invitrogen (A11031)
Alexa Flour 568 goat anti-rabbit IgG (H+L)	Goat	Invitrogen (A11011)
Goat-x-Rabbit IgG-Fc-Fragment-HRP Conjugated	Goat	BETHYL (A120-111P)
Goat-x-Mouse IgG-Fc-Fragment-HRP Conjugated	Goat	BETHYL (A90-131P)

Supplementary Table 3. Primer sequences

Target	Direction	Sequence
Plin2 (mouse)	F	5'-CTCGTCCCTCAGCTCTCCT-3'
	R	5'-TCTCATCACCACGCTCTGTT-3'
I-Fabp (mouse)	F	5'-GTCTAGCAGACGGAACGGAG-3'
	R	5'-TCCTTCATATGTGTAGGTCTGGA-3'
Acox1 (mouse)	F	5'-AATCTGGAGATCACGGGCAC-3'
	R	5'-GAAGTCTTCCCAAGCCCCC-3'
Acox1(human)	F	5'-CGCCTGGAACTTGGAGATCA-3'
	R	5'-AAGTCTTTCCAAGCCCACCA-3'
Elovl6 (human)	F	5'-TGGATGCAGGAAAACTGGAAG-3'
	R	5'-AAGCACCAGTTCGAAGAGCA-3'
Cpt1a (mouse)	F	5'-AGGATACGGTTCGGTCACCA-3'
	R	5'-ACACACCAGTGATGATGCCA-3'
Cpt1a (human)	F	5'-CAAACTGGACCGGGAGGAAA-3'
	R	5'-TGTGCTGGATGGTGTCTGTC-3'
Ech1 (human)	F	5'-AGGTCTTCTGGAGAGAGATGG-3'
	R	5'-GATGTCCGAAGCCATGTCCA-3'
Acaa1 (human)	F	5'-GAGCTTCTCTCGGCAGTCAT-3'
	R	5'-AGTCTCCGGGATGTCACTCA-3'
Hsd17b4 (human)	F	5'-GCAGTGGCCAACTATGATTCAG-3'
	R	5'-ACCCCGCAAATGAACTCTGT-3'
Acad (human)	F	5'-GGTGGTCTCTACCGCGAAC-3'
	R	5'-TTCGGGAGTCCACCTCTTCA-3'
Ppara (human)	F	5'-GCGGCCCAGGCTGAA-3'
	R	5'-ACCAGATGGTGCTGGTTGTG-3'
Abcd3 (human)	F	5'-CGGCCTGCACGGTAAGAAAA-3'
	R	5'-ACAGCTCGCTCCTTTTTCCC-3'
Abcd3 (mouse)	F	5'-GGAGCACAAGCTAGTCCCTT-3'
	R	5'-TGGCCCCAGATCGAACTTCA-3'
Cd36 (human)	F	5'-TCCTGCAGAATACCATTTGATCCT-3'
	R	5'-TGGTTTCTACAAGCTCTGGTTCTTA-3'
Cd36 (mouse)	F	5'-GCTGTTCTAGAAGTTGCTGCC-3'
	R	5'-TACGTGGCCCGGTTCTACTA-3'
Tmem135 (human)	F	5'-CTCCTCTGTACTTGATTGCAGC-3'
	R	5'-CACCAAAGCCAGGAGTCCAT-3'
Tmem135 (mouse)	F	5'-CACCCCTGTACCTGATTGCAG-3'
	R	5'-CACCAAAGCCAGGAGTCCAT-3'
